# Challenges in Septic Shock: From New Hemodynamics to Blood Purification Therapies

**DOI:** 10.3390/jpm14020176

**Published:** 2024-02-03

**Authors:** Fernando Ramasco, Jesús Nieves-Alonso, Esther García-Villabona, Carmen Vallejo, Eduardo Kattan, Rosa Méndez

**Affiliations:** 1 Department of Anaesthesiology and Surgical Intensive Care, Hospital Universitario de La Princesa, Diego de León 62, 28006 Madrid, Spain; jesusmanuel.nieves@salud.madrid.org (J.N.-A.); egarciav@salud.madrid.org (E.G.-V.); carmen.vallejo@salud.madrid.org (C.V.); rmendezh@salud.madrid.org (R.M.); 2 Departamento de Medicina Intensiva del Adulto, Facultad de Medicina, Pontificia Universidad Católica de Chile, Marcoleta 367, Santiago 8320000, Chile; e.kattan@gmail.com

**Keywords:** sepsis, septic shock, hemodynamic coherence, sepsis phenotypes, adsorption therapies, endotoxin, cytokine, blood purification, OXIRIS, fluid tolerance, cardiac dysfunction, sepsis nutrition, hemodynamic monitorization

## Abstract

Sepsis and septic shock are associated with high mortality, with diagnosis and treatment remaining a challenge for clinicians. Their management classically encompasses hemodynamic resuscitation, antibiotic treatment, life support, and focus control; however, there are aspects that have changed. This narrative review highlights current and avant-garde methods of handling patients experiencing septic shock based on the experience of its authors and the best available evidence in a context of uncertainty. Following the first recommendation of the Surviving Sepsis Campaign guidelines, it is recommended that specific sepsis care performance improvement programs are implemented in hospitals, i.e., “Sepsis Code” programs, designed ad hoc, to achieve this goal. Regarding hemodynamics, the importance of perfusion and hemodynamic coherence stand out, which allow for the recognition of different phenotypes, determination of the ideal time for commencing vasopressor treatment, and the appropriate fluid therapy dosage. At present, this is not only important for the initial timing, but also for de-resuscitation, which involves the early weaning of support therapies, directed elimination of fluids, and fluid tolerance concept. Finally, regarding blood purification therapies, those aimed at eliminating endotoxins and cytokines are attractive in the early management of patients in septic shock.

## 1. Introduction

Sepsis, especially septic shock, continues to be a black box in many aspects, and its management poses a challenge for clinicians due to its high mortality—greater than 40% in the case of septic shock [1,2].

Despite the efforts invested in research, its essential management (included in clinical practice guidelines such as the Surviving Sepsis Campaign (SSC) [3]) encompasses hemodynamic resuscitation, antibiotic treatment, life support, and focus control [4].

However, there are aspects that have changed. In hemodynamic resuscitation, influenced by Rivers’ therapy-based packages of measures [5], there is increasing focus on the importance of perfusion and hemodynamic coherence, aided by the universalization of ultrasound as a hemodynamic monitor in intensive care units (ICUs) [6].

In contemporary antibiotic therapy, there are also cases of administration being refined under the umbrella of stewardship programs, the emergence of new antibiotics in a multi-resistance context, and the rapid decrease in bacterial load from a pathophysiological perspective [7,8,9].

There is also increasing emphasis on the importance of organization and specific care programs for sepsis, undoubtedly influenced by packages of measures such as Rivers’, which in isolation may not improve results but do show improvement in terms of cultural change, in addition to the influence of therapies that are sometimes not given the credit they deserve regarding the improvement of results, as is the case of nutrition [10,11].

On the other hand, since it is very difficult to obtain quality evidence in terms of patients with refractory shock (those with higher mortality), the guidelines still lack promising therapy recommendations, such as endotoxin and cytokine elimination therapies through hemoperfusion, that are frequently used [12].

In this narrative review, we attempt to highlight some of the aspects that pose a challenge, as well as current and avant-garde methods of handling patients in septic shock. We focus mainly on hemodynamics (from its most fundamental and innovative aspects), nutrition, and blood purification therapies, all from the perspective of personalization and improving organization-based care [13,14].

The objective is to present a point of view regarding the care of patients in septic shock based on the experience of this study’s authors and the best available evidence to defend their arguments, understanding that the complexity of sepsis should not confine clinicians to a single mental model of care, but rather open them to possibilities in a context of uncertainty [15,16].

The challenges related to sepsis have to do with the balance between evidence-based guidelines and a personalized approach in certain contexts. The high mortality rate of septic shock obligates us to explore cutting-edge therapies [17].

## 2. Improvement Programs in Sepsis: Early Recognition and Rescue

Identifying at-risk patients before they severely deteriorate in the ward presents a great opportunity to improve hospital safety over the next 10 years.

In sepsis, this is important because focus control, resuscitation while there is hemodynamic coherence, and early administration of optimal antibiotic therapy are the keys to success; the sum of these being the best strategy to avoid the need for rescue treatments, progression to a chronic critical illness, or death.

In the latest SSC guidelines, the first strong recommendation is to improve the organization of care for patients with sepsis and the recognition of deteriorating and high-risk patients [3]. There is evidence that performance improvement programs on compliance with sepsis reduce mortality [10,18].

The performance improvement programs known as “Code Sepsis” (SC) have several models for improving care [19,20].

What we propose is a SC conceived and executed as a set of clinical, organizational, analytical, and microbiological tools that, added to intense training and cognitive aids, aim to improve care for sepsis patients, prioritize their care, and refine their treatment.

Following the philosophy of Peter Pronovost in his book *Safe Patients*, *Smart Hospitals*, the implementation of a CS in a hospital must be adapted to its context and must also change its culture if it is to remain and be effective. It should not depend exclusively on a small group of people or personal effort; it should be transversal, perceived as effective, based on training, involve management, and integrate into the culture of the hospital [21].

A published example of this CS model is that of the Hospital Universitario de La Princesa, known as the Princess Sepsis Code (PSC) [22]. A retrospective analysis of PSC patients was carried out between 2015 (the year of the hospital’s PSC implementation) and 2018 to investigate whether the application of this model of care to patients with sepsis could improve outcomes. A total of 1121 patients were included. Mortality showed a statistically significant linear downward trend from 24% in 2015 to 15% in 2018. The application of this model and other models of care to patients with sepsis can result in an improvement in care, even superior to that of medical interventions with evidence.

In the context of sepsis, we want to highlight the concept of rescue in the ward, the scales for detecting deterioration, as well as personalized monitoring.

Failure of rescue in the ward can be defined as an inability to detect clinical deterioration, which leads to the development of severe complications and unplanned ICU admissions. It is a key element in measuring the quality of [23,24]. Hospitals with the fewest rescue failures have the best mortality outcomes, regardless of having the same number of complications [25,26].

Recognition scales for clinical worsening such as quick-SOFA (qSOFA) and National Early Warning Score (NEWS) are increasingly used. In the PSC model, we use qSOFA because of its simplicity and transversality; also, it does not depend on analytics or monitors and incorporates respiratory rate in a prominent way. The most important aspect regarding the use of the different scales is their implementation in the daily clinical routine of hospitalization wards and their relationship with rapid response teams. Regardless of the system used, all staff should be aware of the alert criteria based on the scale and response provided to each of them. Likewise, studies using artificial intelligence and machine learning to create predictive models of sepsis are exponential. Semiautomatic or automatic warning systems, combined with a cultural change in an organization, can be sophisticated but simple and effective tools to facilitate rescue [27,28].

The monitor should follow the patient, not the patient the monitor. The technology already allows for personalized and remote monitoring, which will change the relationship with critically ill patients in the near future and improve the recognition of patients at risk of deterioration [29,30].

## 3. Dynamic Resuscitation in Sepsis in the Context of Fluid Tolerance

Septic shock is a highly lethal disease [31]. Circulatory dysfunction and tissue hypoperfusion are the two key determinants in the development of multiorgan failure and death [32] Early hemodynamic resuscitation can break this vicious cycle, also taking into account that excessive or inadequate therapies can perpetuate organ failure [6].

Hemodynamic resuscitation in septic shock is a dynamic process [13]. This heterogeneity is determined not only by patient-level factors, such as chronic organ dysfunction, infection site, or immune status [6], but also by process-of-care-related factors, such as timely identification of the disease, adequacy of infection control, and provision of life support, among others. This high variability provides a myriad of challenges to clinicians, including rapid diagnostic workup and pattern identification, providing initial care [3], and avoiding fixation errors during the evolution of the disease.

Vincent and de Backer described a temporal paradigm to structure hemodynamic resuscitation in septic shock patients. The acronym SOSD represents the different phases of this process, namely, salvage, optimization, stabilization, and de-escalation [33]. This mental model provides clinicians with approachable endpoints to pursue at each phase, considering the availability of therapeutic interventions and basic and advanced monitoring, kinetics of disease, and complexity of care.

Initially, in the salvage phase, clinical characteristics such as altered mentation, skin perfusion, and low blood pressure can aid clinicians in identifying septic shock patients and prompt resuscitation [34]. Usually, this phase occurs outside of the ICU, either in the emergency room, operating theatre, or ward. Fluids and vasopressors can be administered to restore mean arterial blood pressure and maintain organ perfusion pressure [35]. Clinical signals such as diastolic blood pressure or the diastolic shock index could further aid in determining which patients could benefit from early vasopressor therapy [36].

Once in the ICU, where life-sustaining therapies such as mechanical ventilation are available, further tools can be used to optimize macrohemodynamic variables to restore tissue perfusion [6]. A multimodal approach including basic and advanced hemodynamic monitoring (including central venous pressure (CVP), preload responsiveness tests, and cardiac output (CO) measurements) [37], metabolic parameters (such as central venous oxygen saturation (SvcO_2_), venoarterial carbon dioxide gradient (DpCO_2_), and lactate), and ultrasound-based assessment [38] can further guide fluid, vasopressor, and inotropic support during the optimization and stabilization phases [39].

The final phase comprises the de-escalation phase, which presents its own challenges and opportunities, such as in optimal fluid removal [35]. Both macrohemodynamic and organ perfusion parameters should be monitored in this phase [40]. Some authors have promoted aiming at preload responsiveness and skin perfusion to provide optimal weaning of invasive life support such as mechanical ventilation, but further research should be conducted in this scenario [41,42,43].

This rather linear approach to resuscitation phases has been recently complemented by the novel concept of fluid tolerance [44]. This paradigm integrates monitoring the potential deleterious effects of fluid resuscitation during the earlier phases of resuscitation (salvage and optimization) to avoid side effects and a prolonged de-escalation phase [45]. Thus, alternative resuscitation strategies could be deployed whenever venous congestion signals are detected during the resuscitation process. Specific signals that could aid in this process have yet to be identified, but considering their correlation with adverse clinical outcomes, ultrasound-derived congestion signals such as lung B-lines [46] or VExUS score [47], extravascular lung water indexes [48], and elevated filling pressures could have a role in further guiding fluid administration and avoiding fluid-induced harm [49]. Even though this may seem intuitive, there is rather scant evidence in the current literature, thus making it a rich field for research. Future studies should assess the prevalence of these signals during early phases of resuscitation, the coexistence of fluid responsiveness and fluid intolerance signals, which of these signals carries more risk of adverse events [50], and their integration into resuscitation algorithms.

## 4. Fluid Responsiveness in Sepsis

Fluid response is a physiological condition of the cardiovascular system whereby an increase in preload induced after the administration of a bolus of fluids causes an increase in CO greater than 10% [51].

In the initial phase of septic shock, only 50–60% of patients respond to fluids. After initial resuscitation with fluids, about 25% of patients stop responding to fluids. In light of these data, a volume overload (with its well-known deleterious effects) is possible in a large number of patients [6].

Evaluating the response to fluids is essential in current clinical practice since they should only be received by patients for whom the desired clinical and hemodynamic response will be obtained. It would only be not indicated to determine the response to initial volume in cases where hypovolemia is evident, interrupting its administration when the patient ceases to be responsive to fluids [52].

Although tests to assess volume response have limitations, it is possible to determine it in more than 80% of patients [53].

**Figure 1:**
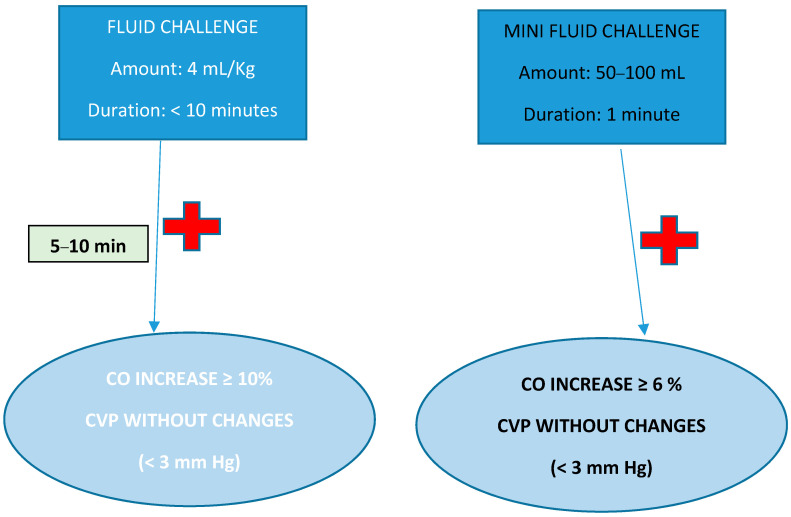
Fluid challenge: implementation, positive fluid response. CO: cardiac output; CVP: central venous pressure.

Three approaches have been proposed to assess fluid responsiveness:

(1) Fluid challenge: This consists of administering small amounts of volume (4 mL/kg) for a short period of time (<10 min), monitoring the effects produced in CO or derived variables after 5–10 min [54] The patient is considered to be fluid responsive if there is a ≥10% increase in CO with minimal variations in the CVP (<3 mmHg). “Mini Fluid Challenge”: Smaller amounts of volume (50–100 mL) are used for 1 min with a 6% increase in CO > 6% [55] (Figure 1).

(2) Fluid responsiveness: The evaluation of volume response using physical or mechanical maneuvers in order to utilize potentially recruitable venous return volume without the need to administer fluids. Examples of this approach are as follows [56,57]:

– Passive leg raising: reproduces the hemodynamic effects of a bolus of 300 mL volume, with a patient with a 10% increase in ≥CO being a volume responder [58].
– End expiratory occlusion test: Consists of interrupting mechanical ventilation for a few seconds, observing the effect produced on OC. With this technique, the expected thresholds in CO variation are lower. A 5% increase is considered a volume response.
– Tidal volume (Tv) challenge from 6 to 8 mL/kg ideal weight for 1 min in patients undergoing mechanical ventilation. It is considered positive when CO increases ≥3.5% [59].
– Cyclic variations: stroke volume variation (SVV) or pulse pressure variation (PPV) during the respiratory cycle.

All of these valuable techniques, even when used in the right context, still have certain limitations.

(3) Ultrasound assessment of variations in superior vena cava (SVC) has been shown to be more reliable than variations in inferior vena cava (IVC), with fewer limitations, but requires a higher degree of invasiveness, as transesophageal echocardiography is required [60].

## 5. Macrohemodynamic Targets; Diastolic Blood Pressure; Vasoconstrictors: Norepinephrine and Vasopressin

(A) Macrohemodynamic goals:

During the initial phase of resuscitation in sepsis, efforts are focused on two goals: restoring mean arterial pressure (MAP) and CO to maintain self-regulation mechanisms, and life-sustaining measures. The optimization phase focuses on improving oxygen transport to the tissues and thus recovering organ perfusion [61].

– MAP: The primary goal of resuscitation should always be tissue perfusion. Under normal conditions, the distribution of blood flow is controlled by metabolic demand through a self-regulating mechanism that is able to divert flow to organs or systems with high oxygen demand. In patients with sepsis, due to the high heterogeneity of organ and system involvement, together with the inflammatory state in which they are found, these mechanisms fail and perfusion becomes more dependent on pressure, which depends directly on MAP [62]. The SSC recommends an MAP of 65 mmHg as the lowest initial intervention threshold. From this threshold onwards, it should be individualized according to each patient [3,63].

The perfusion pressure of the organ also depends on the CVP and interstitial pressure. To select the optimal MAP, the CVP should be considered in conjunction with the associated comorbidities [64].

– Diastolic arterial pressure (DAP): One of the main mechanisms of hypotension and hypoperfusion in septic shock is decreased vasomotor tone. In these cases, DAP reflects vasodilation better than systolic arterial pressure (PAS) or MAP. The severity of vasodilation could influence therapeutic decisions, such as the early introduction of vasoactive agents, which would theoretically avoid unnecessary fluid administration and rapidly restore tissue perfusion [65].

Attention to vascular tone is vital for many reasons, but three points should be considered when addressing patients’ hemodynamic profiles: first, minimal perfusion pressure is likely to be required for adequate organ and tissue perfusion; second, when arterial tone and ventricular systolic function are optimally combined, ventricular performance and efficiency are maximized; and third, the use of DAP together with the diastolic shock index (DSI) can optimize the early use of vasopressors (DSI = heart rate/DAP). Low DAP (especially in the presence of tachycardia), reflecting decreased vasomotor tone, should lead to early initiation of vasopressors even in the absence of central venous access [66].

DAP should be assessed along with heart rhythm. Acute reductions in blood pressure are compensated by an increase in sympathetic activity, although sometimes, such compensation results in a maladaptive response. Since DAP depends on vascular tone and the length of the cardiac cycle, a combination of DAP and heart rate (HR) may reflect the severity of circulatory dysfunction during maladjustment. The DSI could be used as a trigger to target therapeutic interventions in septic shock or sepsis-related cardiovascular dysfunction. A DSI of >2.2 is associated with higher mortality in septic shock; the higher the value obtained, the higher the mortality [65].

(B) Use of vasopressors:

For many years, intravenous fluid administration has been used to improve MAP in hypotensive patients. The current SSC recommendation is to administer 30 mL/kg of fluid (a “weak” recommendation based on low-quality evidence) [3]. This recommendation cannot be applied to all patients, especially not those with comorbidities such as cardiovascular dysfunction or chronic kidney disease. On the other hand, numerous studies have indicated that this goal-directed therapy strategy is not superior to other more conservative methods of fluid administration, so there is increasing controversy as to whether this recommendation is appropriate for all patients [67].

✓ What vasopressors do I use?

- Norepinephrine (NE) remains the most widely used vasopressor and the one recommended by the SSC as the first line [3]. Norepinephrine (NE) is both an alpha1- and beta1-agonist, and is therefore able to increase vascular tone and contractility. NE can improve microcirculatory flow by increasing perfusion pressure in hypotensive patients but can also decrease flow due to excessive vasoconstriction in high doses [68].
- The SSC recommends vasopressin (AVP) as a second-line vasopressor. The SSC suggests adding vasopressin to the NE (weak recommendation, low-quality evidence) with the intention of increasing the MAP or decreasing the dose of NE. This could prevent the deleterious consequences of excessive adrenergic load [3]. Vasopressin has a hemodynamic profile beyond its vasoconstrictor effect, related to its regional vasodilator effects at the renal and pulmonary levels that make it very attractive in several scenarios [69].

✓ When do we start?

The SSC recommends initiating NE within the first hour when fluid administration is not sufficient to achieve hemodynamic resuscitation goals. In the absence of central venous access, they suggest starting their infusion peripherally so as not to delay its administration [3].

There are strong arguments that support the early administration of NE [70,71]:

- Early administration could more quickly reverse the associated hypotension, preventing prolonged severe hypotension.
- Early infusion of NE could increase CO through several mechanisms: increasing preload in the early phase of septic shock and decreasing unstressed intravascular volume of capacitance vessels. In addition, NE may increase CO by increasing cardiac contractility.
- Administration of NE can recruit microvasculature and improve microcirculation in cases of severe hypotension through increased organ perfusion pressure.
- Early administration could prevent the detrimental effects of volume overload.
- Finally, early NE administration could improve patient prognosis [72].

There is growing evidence that the early combination of fluids and NE, most likely during the first hour of resuscitation, has potential advantages in rapidly increasing MAP more than fluids alone, thereby achieving better CO in volume-responsive patients. Resetting higher MAP values corrects hypotension and perfusion better than fluids or vasopressors in isolation, decreases the possibility of fluid overload, and leads to lower morbidity and mortality in the treatment of patients with septic shock [67].

The SSC proposes initiating AVP in septic shock when the base NE dose reaches 0.25–0.5 μg/kg/min [3]. There is consensus among experts to initiate a second vasopressor in cases of refractory hypotension to prevent the effects of an excessive load of NE (called a “decatecholaminisation” strategy) [73]. High doses of NE can compromise the host’s immune system and promote bacterial growth and can induce myocardial cell injury and oxidative stress [70].

High doses of NE may be associated with an increased risk of mortality. Conversely, early multimodal vasopressor therapy may overexpose the patient to AVP and may also be potentially harmful. The main challenge remains to identify the hemodynamic profiles of patients during the initial phase of resuscitation. From a pragmatic perspective, one option is to consider the kinetics of increasing the dose of NE. Basically, two profiles can be observed in NE dose requirements: a “refractory” profile, which corresponds to the need for an exponential increase in NE doses; and a “controlled” profile with a progressive increase in the dose of NE to a plateau, where toxic levels of NE are not reached. In the refractory profile, the earlier the onset of AVP, the greater the possibility of avoiding skyrocketing doses of AN and exposing the patient to harmful doses of AN [74].

## 6. Hemodynamic Monitoring

The choice of the appropriate hemodynamic monitoring technique depends on a patient’s current phase of shock, the complexity of their condition, and their response to initial treatment. Monitoring provides the information necessary to establish the diagnosis of the type of shock, choose the appropriate treatment, and evaluate the response to it.

The diagnosis and treatment of septic shock exposes the clinician to several challenges:

- The coexistence of different types of shock, which can make differential diagnosis difficult.
- Available hemodynamic monitoring systems have limitations.
- Organ-specific perfusion biomarkers are not available.

Despite these difficulties, monitoring methods are being expanded with the use of bedside ultrasound, the use of dynamic parameters to evaluate fluid response, and the evaluation of tissue perfusion markers that provide promising alternatives in the assessment and management of septic shock [62].

Despite current technological developments, we must not forget an initial evaluation based on medical history and physical examination. A patient’s medical record provides the framework in which the rest of the data should be interpreted. A careful, rapid, and focused clinical examination is recommended for the main target organs of tissue perfusion, such as the brain (altered mental state), kidney (oliguria), and skin (cold, mottled skin, prolonged capillary filling time (CRT)). These signs are very useful in the initial phases of shock because they are good indicators of a decrease in CO [75].

(a) Basic monitoring: Central venous catheter, arterial catheter, and initial echocardiographic assessment. Currently, an echocardiographic approach is preferred for the initial evaluation of shock. It provides information to guide the initial diagnosis and assess the response to initial therapy. It is an excellent tool for assessing the condition of the volume. A major limitation of echocardiography is that the estimation of fill pressures is not very accurate and is more suitable for semiquantitative analyses or sequential measurements, as well as in the absence of continuous monitoring.
(b) Advanced monitoring: If the patient’s response to initial treatment is positive and the shock situation resolves, the use of additional monitoring systems is not necessary. If the response is insufficient or inadequate, respiratory distress syndrome (ARDS) coexists, or right ventricular dysfunction develops, then more advanced hemodynamic monitoring is recommended to assess cardiopulmonary function and guide more delicate fluid management [76].

✓ Noninvasive-to-minimally invasive monitors:

Cardiac output monitoring using pulse contour analysis: LIDCO™ Rapid, Most Care-CO, CNEP, ClearSight/Physical, MASIMO^®^, and FloTrac [77].

Most of them are based on a model that integrates the characteristics of aortic impedance, arterial compliance, and systemic vascular resistances.

Although these monitors perform relatively well in stable patients, in unstable patients or patients on vasoactive drugs, they lose accuracy.

✓ Invasive Monitoring:

The CO determined using the thermodilution curve is considered the “Gold standard”.

– Transpulmonary thermodilution systems:

To partially overcome the complications arising from pulmonary artery catheter (PAC) placement, other systems have been developed that are capable of estimating CO using thermodilution.

In addition to being less invasive than PAC, they maintain good precision and accuracy when calculating CO. In combination with the pulse wave contouring technique, it can provide a continuous reading of cardiac flow measurements [78].

It also makes it possible to estimate the volumes of blood present in the pulmonary circulation and in all cardiac chambers. The extravascular lung water index (ELWI) is a quantitative measure of pulmonary edema. The pulmonary vascular permeability index (PVPi) is a marker of pulmonary capillary leak. Both are independent predictors of mortality in ARDS [48].

Today’s monitors assess the patient’s blood volume by estimating volumes and pressures, as well as the potential consequences of excess volume, such as ELWI and venous congestion. Just as important as determining the need for fluids is diagnosing excess fluids. Cardiac preload indices can be used to detect hypervolemia. It is important to note that the presence of edema does not exclude the need for fluids. Also, a positive water balance is not systematically accompanied by an increase in blood volume, so other variables must be considered. In this regard, measurement of ELWI and venous congestion indices could be useful.

**Figure 2:**
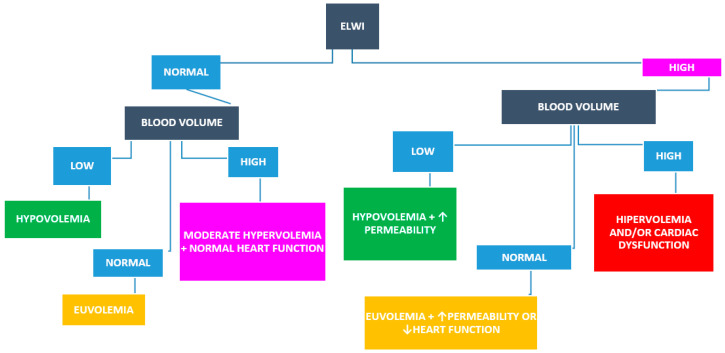
Interpretation of volume status integrated with pulmonary extravascular water index. Modified from de Bakker et al., 2022 [6]. ELWI: extravascular lung water index.

ELWI may be elevated due to an increase in intravascular pressure at the pulmonary level or due to an increase in pulmonary capillary permeability. Increased intravascular pressure may be related to cardiac dysfunction or an increase in central blood volume. Combining volumetric measurements, ELWI, and CVP can be useful in separating the different options. (Figure 2).

## 7. Tissue Perfusion Monitoring: Lactate, Venoarterial Carbon Dioxide Gradient, and Capillary Refill Time

Peripheral tissue hypoperfusion has been identified as a powerful predictor of poor prognosis in patients with sepsis. The use of metabolic parameters for the assessment of regional microvascular perfusion is promising, but not without some limitations. The combined use of these parameters allows us to better assess peripheral tissue perfusion than their use in isolation.

Lactate: Although catecholamine-induced glycolysis and decreased hepatic clearance are important causes of hyperlactacidemia during sepsis, inadequate tissue perfusion is the most important cause of elevation during septic shock and thus a useful biomarker in the diagnosis of septic shock.

Elevated lactate values (>4 mmol/L) at baseline and during the first 24 h have been associated with increased mortality. There is growing evidence that lactate clearance is higher than absolute values and better reflects the effect of therapy. The periods of evaluation of lactate variations have also been assessed, with significant variations in short time periods (20 min) [79].

Although the decrease in lactate after the establishment of initial resuscitation measures is associated with an improvement in outcomes, great care should be taken in interpreting the response of lactate levels to therapeutic maneuvers only in the context of hypoperfusion. It is important to interpret lactate values in conjunction with other perfusion parameters.

Normalization of lactate may be delayed, even if its production is declining due to the resolution of the shock. Other factors, apart from anaerobic metabolism, can increase lactate production. Sustained hyperlactacidemia suggests the need for re-evaluation of treatment, but more precise guidelines on serial lactate measurements are needed to assess response to treatment.

Venoarterial carbon dioxide gradient (DpCO_2_): Tissue CO_2_ represents the balance between local CO_2_ production and removal. A high value probably reflects a decrease in local blood flow, rather than an increase in CO_2_ production. When arterial CO_2_ is associated with tissue CO_2_, it allows for the determination of DpCO_2_, which is inversely related to the proportion of perfused capillaries and thus to CO.

DpCO_2_ has also been shown to be a good indicator of poor prognosis in septic patients. In septic shock, when oxygen uptake (VO_2_) is insufficient due to microcirculatory or mitochondrial alterations, central venous oxygen saturation (ScvO_2_) can reach normal or supranormal values. In such circumstances, DpCO_2_ > 6 mmHg is a good indicator of tissue hypoperfusion; the complementary use of ScvO_2_ and DpCO_2_ is therefore recommended. In the case of a DpCO_2_ > 6 mmHg, one of the therapies would be to increase CO [80].

Capillary refill time (CRT): The skin does not possess circulatory autoregulation mechanisms, so sympathetic activation impairs its perfusion if there is circulatory dysfunction. Alteration of peripheral perfusion indicators is associated with increased mortality and morbidity. CRT has been shown to be a parameter with an excellent profile for use in routine clinical practice. It is a simple parameter, available in all environments, with a fast response time to the maneuvers established and useful at all times of the sepsis process [81].

After initial resuscitation, a prolonged CRT determination longer than 3 s identifies patients with sepsis with worse outcomes, both in the emergency room and in the ICU [82].

In the ANDROMEDA-SHOCK trial, CRT was monitored every 30 min to guide the strategy of early resuscitation in patients with septic shock, and suggested benefits were compared to lactate-based monitoring [83].

An excellent correlation between CRT and the passive leg-raising maneuver has also been described, suggesting a rapid relationship (10–12 min) between this parameter and volume expansion maneuvers. The decrease in CRT after volume administration could reflect improvement in microvascular perfusion, which is the goal of these maneuvers [84].

Other important aspects involved in tissue perfusion are CO and vascular tone. When peripheral tissue hypoperfusion improves without changes in cardiac index after fluid infusion, it may be due to blood dilution or improvement in microvascular rheology.

Due to these characteristics, the use of CRT has been adopted in the clinical guidelines of the SSC, associated with other tissue perfusion parameters [3].

## 8. Hemodynamic Coherence

Hemodynamic coherence is the correct relationship between macrocirculation and microcirculation and should be the objective of all maneuvers performed on patients with septic shock [85].

It is a condition in which resuscitation maneuvers established to correct systemic hemodynamic variables are effective in correcting regional perfusion, microcirculation, and oxygen delivery (DO_2_) to tissues, so that cells can sustain and maintain cellular and organ functionality.

In the last decade, attempts have been made to determine the parameters to guide the use of fluid therapies and vasoactive drugs (NE, inotropics/inodilators, and vasodilators), as well as drugs that act specifically on the endothelium. One of the most widely used is sublingual microcirculation. For example, Dubin et al. found that NE did not improve flow at the microcirculatory level in all patients, but only in those with pre-existing microcirculatory impairment. It is interesting to determine which patients benefit from the therapies in order to be able to individualize the treatments [86].

The main conclusion of these studies was the importance of individualization in resuscitation and the deep interweaving of the concepts of macro- and microcirculation [40].

From a physiological point of view, the value of hemoglobin is decisive for tissue oxygenation, much more so than convective flow. Therefore, the occurrence of dilutional anemia during the resuscitation of these patients must be avoided. A very interesting monitoring target that combines convection, diffusion, and DO_2_ data is tissue red blood cell perfusion (tRBCp). These types of parameters could safely guide transfusion therapy, as well as the use of vasodilator drugs, but there is still a lack of evidence to support their use and to defend their use with clinically relevant results [87].

Bedside microcirculation monitoring is still limited by the lack of objectivity in image interpretation. This limitation could be overcome with the development of software that assists clinicians in the use of these parameters.

## 9. Nutrition in SEPSIS

The assessment and nutritional treatment of patients with sepsis is very difficult, as all inflammatory processes, metabolic and immune changes, and organ dysfunction compromise this process. In addition, low adherence to clinical nutrition guidelines has been demonstrated [88].

It is not as clear whether the acute phase of sepsis is always associated with a hypermetabolic state as traditionally thought. The metabolic response during sepsis progresses in opposite directions, showing signs of excessive inflammation as well as immune suppression. Cellular metabolic processes undergo fundamental changes, without the ability to recover their normal homeostasis. The magnitude of inflammation and immunosuppression varies among patients, and depends on the host, pathogen, and therapy. Energy requirements change with the evolution of the disease and between different patients.

Insufficient nutrition and immune dysfunction do not have a synergistic effect on mortality in critically ill patients with sepsis. As expected, a well-nourished patient with normal immune function has a better chance of survival. However, insufficient nutritional intake in patients with marked immune impairment was not shown to be as harmful as might be expected [89].

- Calories:

Calculating energy requirements by means of predictive formulas often underestimates the actual need and exposes patients to situations of malnutrition.

There is little evidence to suggest that high-calorie intakes do not affect morbidity and mortality. Caloric targets should ideally be individualized by measuring resting energy expenditure with indirect calorimetry, which is becoming increasingly simple and accessible. Nutritional screening in the different phases of the septic process is more appropriate than a punctual approach, or the usual approach using equations.

Any calorie plan should also consider other less controllable factors. For example, endogenous glucose production can cover up to 75% of energy needs in the first 3 or 4 days of critical illness. This is why one must increase calorie intake slowly. Careful monitoring of blood glucose is essential, as abnormally elevated or decreased blood glucose measurements are associated with a significant increase in mortality [90].

The proportion of septic patients included in clinical trials of ICU nutrition is low, and information is often extrapolated from other patient populations.

Studies evaluating the outcomes of septic patients treated with different caloric regimens are still lacking. What the existing studies have shown so far is that nutritional alterations are deleterious both by excess and by defect. High-calorie administration with respect to energy expenditure (greater than 70%) has been associated with increases in mortality, duration of mechanical ventilation, and ICU stay [91].

- Proteins:

The objective of enteral or parenteral protein administration in critically ill patients is to ensure and improve protein synthesis to slow muscle loss and increase muscle regeneration processes. However, the dosage, timing, and risk–benefit ratio of protein supplementation in sepsis remains undetermined. Given the low level of evidence, international clinical nutrition guidelines recommend a wide variety of protein regimens [92].

Current evidence suggests caution regarding the administration of high protein doses in critically ill patients on mechanical ventilation, as they do not improve hospital stay and their administration could worsen the outcomes of patients with renal failure as well as be associated with worse outcomes on organ dysfunction scales [93].

- Enteral vs. Parenteral Nutrition:

Both feeding routes are useful for nourishing septic patients. Apart from the specific limitations and contraindications for each particular route, an early increase in enteral nutrition increases the risk of severe digestive complications in patients with shock, while non-individualized early parenteral nutrition is associated with worse ICU outcomes. Parenteral nutrition continues to be a valid option if enteral nutrition fails to deliver calorie targets after 3 to 7 days of the acute process [94].

Enteral nutrition is cheaper than parenteral nutrition and theoretically represents the most physiological route of feeding. Enteral nutrition improves gastrointestinal blood flow, preserves the intestinal mucosa, stimulates enzymatic processes, and enhances the systemic immune response. An immediate clinical benefit of enteral nutrition in septic patients is the prevention of bacterial translocation and stress ulcers. Enteral nutrition is considered safer than parenteral nutrition because central venous access is not required and its undesirable effects, such as hyperglycemia, hyperlipidemia, fatty liver, etc., are avoided [95].

However, enteral nutrition is not completely risk-free. Nutrition may be suboptimal due to irregular absorption. Difficulties in gastric contents passing through the pylorus (ileus, slowing of gastric emptying, etc.) could increase the risk of aspiration. Transit disorders, such as vomiting and diarrhea, greatly hinder the progression of enteral nutrition and are contraindicated in cases of intestinal damage, either ischemia or obstruction. In patients in shock, enteral nutrition could worsen the risk of intestinal ischemia, as it places a heavy workload on a hypoperfused intestine [96,97].

Refractory shock, severe hypoxemia, and acidosis continue to be contraindications to enteral nutrition according to the clinical guidelines of the European Society of Intensive Care Medicine (ESICM) [98].

- Pharmaconutrition:

Pharmaconutrition consists of the association of nutrients with beneficial actions specific to standard nutrition (e.g., antioxidant effects). Its main goal is to reinvigorate the intestinal mucosa and the defense mechanisms of the immune system, as well as to limit an excessive proinflammatory response during the catabolic phase of the disease.

The most relevant pharmaconutrients in septic patients are the amino acids glutamine and arginine, omega-3 fatty acids, selenium, and vitamin C [99].

The theoretical benefit of pharmaconutrition is not transferred to the level of clinical outcomes in septic patients. The clinical evidence justifying the use of glutamine, arginine, selenium, or fatty acids is weak, and effective supplementation could be deleterious for these patients. Omega-3 fatty acid supplementation is associated with better outcomes in patients with ARDS, but it remains unclear whether this effect depends only on this component or on improvement in all therapeutic measures in the management of ARDS [100].

Despite promising results from different trials and meta-analyses, which showed good clinical results with no difference in mortality rate, routine vitamin C administration is not currently recommended in patients with sepsis. In addition, the optimal dose of vitamin C remains a matter of debate, and dose–response studies are still required. High intravenous doses of vitamin C are considered safe, as potential side effects, such as oxalate nephropathy, pro-oxidant effects, and hypotension, are rare [101].

### 9.1. Current Nutrition Recommendations for Sepsis

#### Nutrition Stewardship

Nutrition should be considered like any other pharmacological treatment, and nutrition stewardship measures should be implemented in ICUs following the “six D’s”: diagnosis, drug, dose, duration, de-escalation, and discharge [11].

Patients should be provided with calories, either enterally or parenterally, guiding the amount by indirect calorimetry, in combination with other methods such as electrical bioimpedance analysis and nitrogen balance. Protein should also be given at doses of 1.3 g/kg/day when symptoms begin to subside, in combination with low doses of glutamine in exclusively parenterally nourished patients, as well as nutrients including carbohydrates, proteins, and lipids [102].

Nutrition should be considered as a drug, with indications and contraindications, potential adverse effects, and specific characteristics. For each type of nutrition, there are specific considerations (pharmacokinetics and pharmacodynamics, volume kinetics, etc.).

The duration of artificial nutrition, whether total or supplemental, is equally important. Doses should be adjusted to the shock phase, decreasing parenteral doses when the shock is resolved and the gastrointestinal tract is functioning normally. Proper maintenance or suspension upon discharge from the ICU or hospital is required as part of the nutritional care plan and should be guided by current quality standards [102,103].

## 10. Myocardial Dysfunction in Sepsis

Myocardial dysfunction or septic cardiomyopathy (SCM) can be defined as acute, potentially reversible cardiac abnormality unrelated to ischemia, which exhibits one or more of the following features [104]:

– Systolic or diastolic ventricular dysfunction of the left and/or right ventricle;
– CO preserved or diminished;
– Arrhythmias.

The pathophysiology is complex and still subject to debate. Among the many factors that have been proposed for the development of SCM, the following stand out [105]:

– Inflammatory and cardiac-depressant molecules: cytokines (especially tumor necrosis factor (TNF) and interleukin-1 (IL-1)), nitric oxide, prostanoids, and lipopolysaccharides (LPS).
– Mitochondrial dysfunction and increased exosome secretion, leading to an increase in reactive oxygen species (ROS) and decreased adenosine triphosphate (ATP) production.

Risk factors for SCM include male sex, young age, hyperlactacidemia, and history of heart failure [106].

When SCM was initially described in the 1980s, it was defined as an acute decrease in left ventricular ejection fraction (LVEF). Initial data suggested that patients with low LVEF and increased left ventricular (LV) size survived longer [107]. However, it has recently been shown that LVEF is not an accurate parameter of extrinsic cardiac function, mainly because it is highly dependent on ventricular loading conditions [108].

For this reason, the diagnosis of systolic dysfunction in sepsis continues to be a challenge. There are new technologies such as ventricular strain, which has proven more sensitive than LVEF for the diagnosis of LV dysfunction in sepsis; however, its routine use in critical care units is not yet widespread due to the need for high-end equipment and software as well as special training [109]. Other echocardiographic parameters have also been evaluated to detect LV systolic dysfunction, such as mitral annular plane systolic excursion (MAPSE), which shares similarities with ventricular strain but is much easier to perform. Combining MAPSE with APACHE II provides a good predictor of mortality in septic patients with SCM [110].

Diastolic dysfunction is also of great importance; it is associated with the administration of fluids in the resuscitation phase of septic patients and with an increase in mortality [111]. Therefore, it is imperative to recognize that patients are at risk of volume overload during the treatment of septic shock.

**Figure 3:**
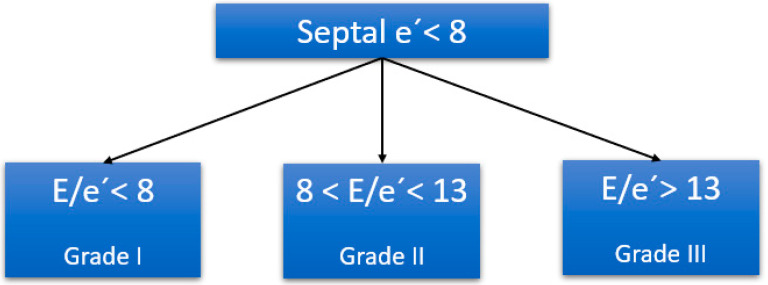
Simplified diastolic dysfunction algorithm. Modified from Lanspa et al. (2016) [113].

Diastolic dysfunction diagnosis based on the 2016 American Society of Echocardiography (ASE) algorithm can be difficult to carry out in critically ill patients [112]. It is for this reason that simplified algorithms have been proposed for septic patients, most notably that of Lanspa et al., in 2016 (Figure 3) [113].

The advantage of this algorithm is that it only requires the measurement of early diastolic flow (E) by pulsed Doppler and the measurement of the early diastolic velocity of the middle septal and lateral mitral ring (e′) obtained by tissue Doppler. With these two values, the E/e’ ratio is obtained, which is capable of categorizing more patients with diastolic dysfunction compared to the algorithms of the 2009 and 2016 ASE guidelines, in addition to presenting a reasonable correlation with relevant comorbidities of septic patients [114]. On the other hand, the E/e’ ratio can assist clinicians in making decisions such as volume management or weaning from mechanical ventilation [115].

### Cardiac Biomarkers in Sepsis

Brain natriuretic peptides (BNPs) such as N-terminal pro-B-type natriuretic peptide (NT-proBNP) are secreted by myocardial cells in response to myocardial stretching. This would theoretically make NT-proBNP a useful biomarker for the diagnosis of systolic and diastolic ventricular dysfunction in sepsis. However, elevated levels of NT-proBNP in septic patients may have more causes than SCM, such as congestive states, useful for their management [116].

Pulmonary involvement (respiratory infection, ARDS, mechanical ventilation), previous heart failure, and renal failure are among the etiologies associated with elevated NT-proBNP in sepsis [104]. However, NT-proBNP can behave as a prognostic marker in critically ill patients, including septic patients. A 2020 meta-analysis found that NT-proBNP >4000 pg/mL has a sensitivity of 72% and specificity of 78% in predicting mortality in septic patients [117]. The authors of this study acknowledge that the results should be interpreted with caution due to the great heterogeneity of the patients included in the analysis.

## 11. Role of Levosimendan in Sepsis

Levosimendan is a cardiotonic drug that improves the sensitivity of troponin C to calcium in myocardial cells (thereby improving contractility), with little effect on myocardial oxygen consumption.

Levosimendan has been shown to improve the hemodynamic profile of patients with septic shock and SCM [118,119]. In a recent randomized controlled trial (RCT) of patients with septic shock and LVEF < 35% (n = 30), the authors found that levosimendan improved myocardial contractility, decreased catecholamine requirements, and reduced myocardial damage and mechanical ventilation time without any impact on mortality [120].

The SSC suggests the use of dobutamine as the inotropic agent of choice, dobutamine plus norepinephrine or epinephrine in isolation in septic patients presenting with cardiac dysfunction, and persistent hypoperfusion after initial resuscitation (i.e., after adequate volume and blood pressure status have been achieved). With respect to levosimendan, they suggest against its use in this context (both weak recommendations and low quality of evidence) [3]. The rationale for this recommendation against levosimendan is based on two studies: The first, the 2016 “leoPARDS” trial, enrolled 515 patients with septic shock (without discriminating the presence or absence of SCM) who were randomized to receive levosimendan vs. placebo. The use of levosimendan was found to not decrease mortality, increase norepinephrine requirements, decrease extubation success, and increase the rate of supraventricular arrhythmias [121]. The second study is a 2017 meta-analysis comparing seven RCTs of levosimendan vs. dobutamine and finding no difference in mortality, although patients treated with levosimendan had lower serum lactate levels and higher cardiac indices [122].

In addition to what is described above, the SCC recommendations add cost and limited availability, especially in low-resource scenarios (based on the principle of equity), to their recommendation against the use of levosimendan.

In our opinion, we advocate the use of levosimendan in patients with SCM whenever available, due to its pharmacological profile. The treatment of sepsis is based on bundles of measures that, together, improve the chances of survival. In this case, we believe that levosimendan is superior to dobutamine and adrenaline in improving the hemodynamic profile of septic patients and that this might increase the chances of recovery, especially in patients with associated diastolic alteration and/or signs of cardiac congestion [123,124].

## 12. Role of Echocardiography in the Diagnosis, Resuscitation, and Management of Septic Patient Congestion

The role of echocardiography in the diagnosis of patients in shock has been increasing for more than a decade [125]. The role of ultrasound in these patients can be divided into three main sections:

(1) Differential diagnosis of the patient in shock;
(2) Initial resuscitation of the patient with septic shock;
(3) Managing the congestion or “evacuation” phase of sepsis.

### 12.1. Differential Diagnosis of Shock

There are several echocardiography-based algorithms for the assessment of shock in critically ill patients [126]. In a 2023 meta-analysis that included 1132 patients with shock, echocardiography, and specifically point-of-care ultrasound (POCUS), was found to have a sensitivity, specificity, and area under the curve (AUC) of 0.79, 0.96, and 0.86, respectively, to differentiate distributive shock, in addition to similar values for the rest of the subtypes of shock except obstructive, where it was slightly higher [127].

**Figure 4:**
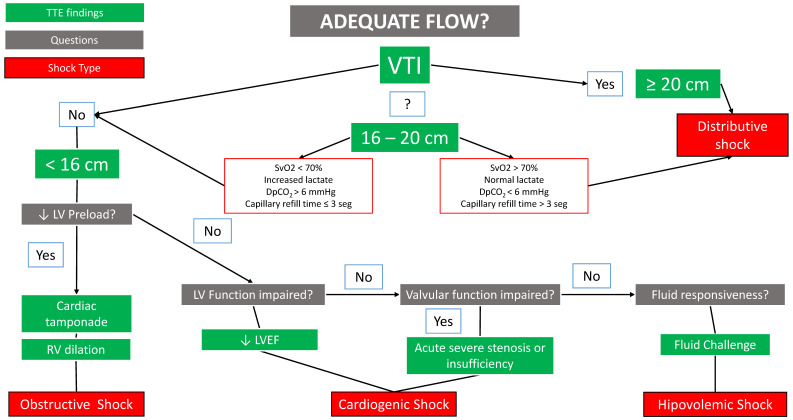
Perfusion-based differential diagnosis algorithm. Modified from Mercadal et al. 2022 [120]. DpCO_2_: venoarterial carbon dioxide gradient; LV: left ventricle; LVEF: left ventricular ejection fraction; RV: right ventricle; ScvO_2_: central venous oxygen saturation; TEE: transesophageal echocardiography; VTI: velocity time integral.

Recently, Mercadal et al. proposed a new algorithm that is easy to learn and apply and may be useful for the differential diagnosis of shock (Figure 4) [128]. This algorithm focuses on perfusion parameters to differentiate the type of shock, the main one being the velocity time integral (VTI) of the left ventricular outflow tract (LVOT).

In summary, the algorithm initially evaluates LVOT VTI. If the patient’s VTI is >20 cm, distributive shock is present, and in case of suspected infection, septic shock is the first possibility. If VTI is <16 cm, the rest of the causes of shock are ruled out in the following order: obstructive, cardiogenic, valvular cardiogenic, and hypovolemic. If VTI is between 16 and 20 cm, other perfusion variables such as SvcO_2_, lactate, DpCO_2_, or CRT should be used to reach the diagnosis. This range of 16 to 20 cm is the so-called grey area.

When interpreting the results, the authors of the algorithm give the following warning:

– The order of the algorithm is not trivial: it is designed to avoid volume overload (for this reason, the last type of shock to be ruled out is hypovolemic).
– Several shock mechanisms can coexist in the same patient, so the one considered the most predominant should be treated first, and the algorithm should always be completed to the end.
– The algorithm does not cover 100% of shock causes.

### 12.2. Cardiac Ultrasound to Guide Resuscitation and Initial Management of Sepsis

Geri et al. identified five echocardiographic phenotypes in septic patients during the first twelve hours after diagnosis and after the initial administration of volume and vasopressor drugs [129]. To obtain the results, they used combined information from two databases with a set of 360 patients with septic shock admitted to the ICU who underwent transesophageal echocardiography (TEE). From a methodological point of view, they used a clustering approach that allowed them to differentiate five different types of patients with septic shock from an echocardiographic and hemodynamic point of view (Table 1):

– Cluster 1 “Well resuscitated”: patients with preserved bilateral ventricular function and no fluid responsiveness.
– Cluster 2 “LV systolic dysfunction”: compromised systolic function, low CO, higher dose of vasopressors, and no fluid responsiveness.
– Cluster 3 “Hyperkinetic state”: preserved ventricular function, elevated CO, and no fluid responsiveness.
– Cluster 4 “RV failure”: RV dysfunction and dilation, hypoxemia, and no fluid responsiveness.
– Cluster 5 “Still hypovolemic”: preserved ventricular function, low CO, and fluid responsiveness.

These phenotypes can help clinicians guide therapy after the initial resuscitation of septic patients, for example, by identifying which patients are likely to require more volume (Cluster 5), who benefits from inotropic support (Cluster 2), potential candidates for the use of beta-blockers (Cluster 3), or even identifying patients at risk of developing ARDS (Cluster 4).

In addition, it is possible to establish a prognosis based on the phenotype. Of the five ultrasound patterns studied, two of them (Clusters 1 and 3) are associated with ICU mortality around 20%, while the rest (Clusters 2, 4, and 5) are associated with ICU mortality around 40%.

It should be noted that the results have not been validated prospectively or by the use of transthoracic echocardiography (TTE). In addition, there is some overlap between clusters, as the authors acknowledged in the limitations of the study.

### 12.3. Volume Overload and Venous Congestion

During the treatment of critically ill patients with sepsis, fluid administration is one of the fundamental components, along with focus control and antibiotic administration. Volume overload in these patients is associated with worse outcomes and elevated mortality [130].

In recent years, the focus has been on the amount of volume administered, and perhaps just as important when and how to start evacuating that volume. In this regard, Malbrain et al. divided sepsis into four phases that they called ROSE (resuscitation, optimization, stabilization, and evacuation) [35]. The “E” phase, evacuation, involves the exit of fluids accumulated spontaneously or forcibly (diuretics or renal replacement therapy), so the questions, “When to start fluid removal?” and “When to stop fluid removal?” arise.

**Figure 5:**
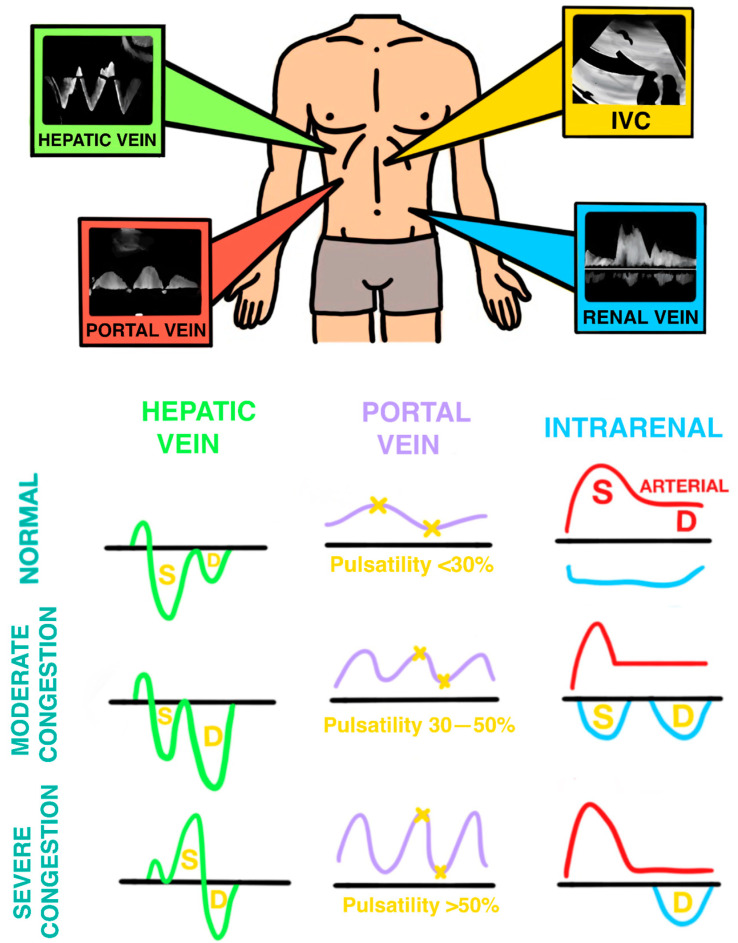
Venous excess Doppler ultrasound (VExUS) diagram.

A new ultrasound tool described by Beaubien-Souligny et al. in 2020 could help answer these questions. This is a systemic congestion classification system called venous excess Doppler ultrasound (VExUS), based on POCUS [47]. VExUS classifies venous congestion of the abdominal organs as normal, mild, or severe by examining the following patterns (Figure 5):

– The diameter of the inferior vena cava and its collapsibility.
– Flow from the suprahepatic veins.
– Portal vein flow.
– The flow of intraparenchitmatous renal veins.

VExUS was initially validated in the postoperative period of cardiac surgery, where the presence of severe congestion was associated with the occurrence of acute renal failure (AKI) with a hazard ratio (HR) of 3.69 ((CI: 1.65–2.84), *p* = 0.001) [47]. Similarly, an association was found between elevated VExUS and AKI development in patients with acute coronary syndrome [131]. However, in a cohort of patients admitted to a general ICU (n = 145), no significant association was found between the grade of VExUS and the occurrence of AKI or mortality [132].

It is plausible that in septic patients, once resuscitated and stabilized, VExuS may help determine when to initiate fluid evacuation, as well as help guide it.

So far, no studies on septic patients have been published, although, to our knowledge, at least two are being conducted: First, the randomized clinical trial ANDROMEDA 2, accompanied by a sub-study of VExUS that aims to find the association between the score and outcomes in septic patients [14]. Second, Romano et al. are working on a prospective observational study protocol that adds lung ultrasound (detection of pulmonary congestion by quantification of B-lines) to VExUS, named VExLUS, with the aim of increasing sensitivity and precocity in the detection of fluid overload and guiding clinicians on when to stop fluid administration [133].

## 13. Blood Purification Therapies in Sepsis

### 13.1. Overview

Blood purification therapies are showing great development and growing popularity in sepsis due to improvements and technological advances, but above all, due to the conviction of a part of the medical community of their usefulness in septic shock [134].

Renal replacement therapies can also be considered a form of blood purification, since they are often used to eliminate waste or toxic products that, due to acute kidney injury (AKI), cannot be eliminated by the kidney [135]. However, in this part of the article, we refer more to the purification of the molecules responsible for the disproportionate and/or aberrant response of the host to the infection, such as endotoxin, pathogen-associated molecular patterns (PAMPs), danger-associated molecular patterns (DAMPs), and cytokines [136].

The management of sepsis is based on hemodynamic resuscitation according to phenotypes, with optimal life support, early focus control, and infection treatment with antibiotic therapy [13,137].

Renal purification therapies can play an important role in two phases, acting on the pathophysiology of the infection and being a life-sustaining treatment [138].

In sepsis, local invasion by microorganisms and their dissemination occurs; the immune system response is aberrant and produces organ failure:

1. Invading microorganisms generate PAMPs, which are small sequences of molecules that repeat in groups of pathogens. The most well-known is endotoxin expressed on the surface of Gram-negative bacteria (GNB).

2. These PAMPs are recognized by receptors on innate immune cells, such as neutrophils, which trigger the release of inflammatory mediators by those cells, the most important of which are cytokines.

3. Cytokines can cause, among other actions, cell destruction, and these injured cells produce DAMPs. Like PAMPs, they also have receptors on immune cells, again generating more cytokines and mediators of inflammation, and thus perpetuating a vicious cycle of uncontrolled immuno-inflammation that causes what we call sepsis.

4. Through these mediators, sepsis causes vasoplegia, endothelial dysfunction, coagulation disorders, and organ failure, including very common kidney failure. (Figure 5) [139].

The SSC’s recommendations are based on the GRADE methodology [3]. Their philosophy includes not recommending if there is no evidence, assessing risk–benefit, and not recommending if it may be difficult to afford, such as in places with fewer resources. We must therefore understand the recommendations of the SSC regarding blood purification therapies with this approach, whereby, due to the high price of the treatment and the absence of clear scientific evidence in its favor, the recommendation is against the use of polymyxin (weak recommendation and low quality of evidence) and without evidence to make a recommendation in the case of other blood purification techniques. Despite these recommendations, its use is becoming more and more widespread, based on the pathophysiological mechanism of sepsis itself and on clinical practice with excellent results in the centers where these therapies are used [139].

An effort should be made to personalize and focus these endotoxin and cytokine hemadsorption therapies within precision medicine, as we report in the rest of the sections of this article [140].

Individualization in ICUs is necessary, and the guidelines should be understood in this context, considering that blood purification therapies are among the most promising in this personalization effort and that they may benefit from diagnostic enrichment strategies in sepsis in the near future.

### 13.2. Rational of Blood Purification Therapies in Sepsis

To understand how blood purification therapies can improve the treatment of sepsis, it is essential to understand the pathophysiology of sepsis, and the reason for the failure of dozens of treatments aimed at controlling only some of the molecules and mediators involved in it [141].

**Figure 6:**
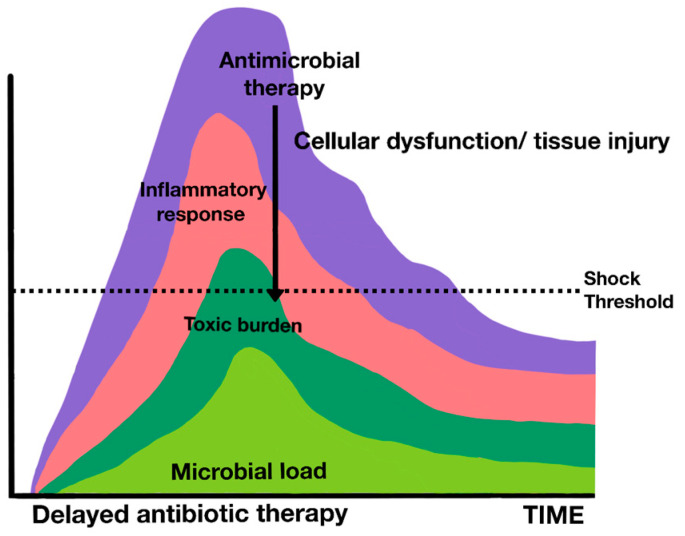
Delayed antibiotic therapy.

**Figure 7:**
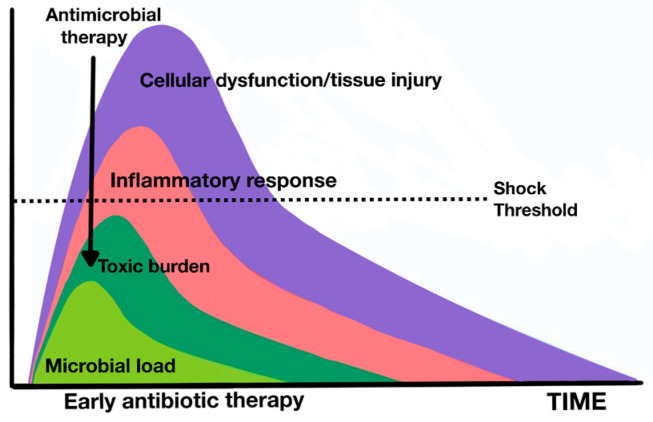
Early antibiotic therapy.

It is interesting to highlight the pathophysiological theory of Anand Kumar, which highlights the importance of bacterial load and endotoxin in the prognosis of patients in septic shock [142]. Kumar, beyond the immunologically or microbiologically restricted pathophysiological view, proposed an alternative pathophysiological model, with a substantial implication of bacterial load and endotoxin (Figure 6 and Figure 7).

This load would be the main driver of organ dysfunction and should therefore be the goal of the patient’s therapy. The key to improving results would be to reduce this bacterial load as soon as possible, by optimizing antibiotic therapy and implementing all strategies for this purpose, such as the adsorption of endotoxins and cytokines. Therefore, precocity is fundamental in this model, as the concept of irreversible shock comes into play. In this model, shock, regardless of its etiology, can only be tolerated for a limited time. Once established, it becomes irreversible, triggering an inevitable progression toward death or multiorgan failure and chronicity in intensive care in the best of cases, unless it is reversed within a short period of time.

This theoretical model also suggests that septic shock and sepsis are two different entities rather than a continuum of the same disease. The main difference between them lies in the time available until the onset of irreversible and irreplaceable organ failure. The simple evidence of observation of clinical behavior (hypotension, lactic acidosis, and fatigue of compensatory responses), added to the large differences in mortality between septic shock (greater than 40%) and sepsis (a more moderate clinical entity with lower mortality), invites us to think of these two entities as different, according to Kumar [142].

In this review, we focus on GNB sepsis. As the intestinal microbiota contains a large amount of GNB and therefore endotoxin, if bacterial translocation occurs, in many situations, we can find endotoxemia, which produces shock and poor perfusion [143].

When GNBs produce an infection at the local level, they generate a response and damage activated by innate immunity, which responds to the recognition of PAMPs, generating a defensive response that may or may not be proportional. PAMPs include, among others, the lipopolysaccharide of the GNB cell membrane or endotoxin, especially its most active part, lipid A.

This PAMP is best known for its clinical significance and can be acted upon by reducing its load using blood purification therapies. If the amount of endotoxin is not excessive, it does not pass the shock threshold, and can be assumed by immunity, generating an adequate and proportionate immune response. If the response generated by PAMPs is excessive, what we know as septic shock begins with vasoplegia, myocardial dysfunction, coagulopathy, encephalopathy, renal failure, and alteration to the intestinal barrier, among others.

Innate immunity recognizes these patterns through specific receptors, such as Toll-like 4 (TL4). As a consequence of the activation of these receptors and the response they trigger, macrophages, neutrophils, endothelial cells, and the coagulation cascade are activated [144,145].

**Figure 8:**
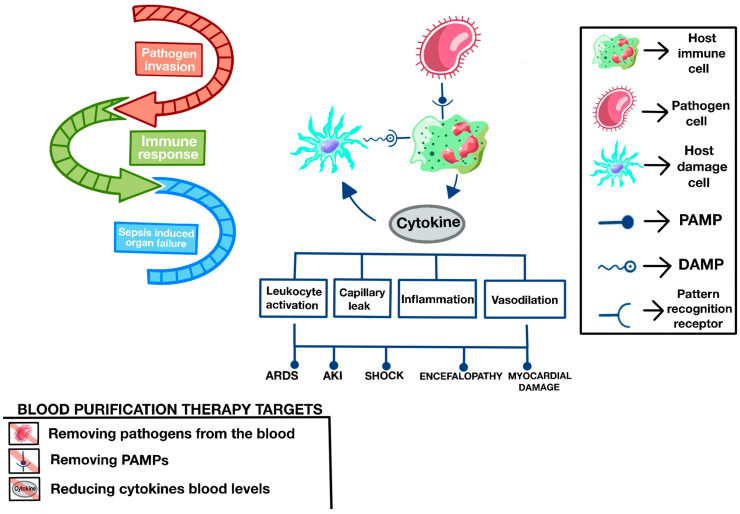
Mechanisms of pathological immune response to infection in sepsis, their relationship with organ failure, and the targets of blood purification therapies.

The cytokines in the first phase are proinflammatory (IL 6, IL1, TNFα, etc.), and in the second phase are anti-inflammatory (IL 10, TGFβ, etc.). These cytokines are responsible for the pathophysiology associated with sepsis, endothelial damage, and coagulation activation [140]. The damage associated with cell destruction generates other molecules (DAMPs), which are also recognized by immune cells, generating even more cytokines and mediators, thus entering a vicious cycle that is perpetuated (Figure 8) [143].

In 2011, the Nobel Prize in Physiology was awarded jointly to Bruce A. Beutler and Jules A. Hoffmann for their discoveries on the activation of innate immunity and the role of TL4s. This provides us with perspective on its importance [146].

PAMP: pathogen-associated molecular pattern; DAMP: danger-associated molecular pattern; ARDS: acute respiratory distress syndrome; AKI: acute kidney injury.

Currently, there are therapies that target these different phases and that can be applied individually or in association with others in a synergistic way. Sepsis trials of therapies targeting molecular mediators and patterns have been conducted with unsuccessful results. In these trials, the blockade was of only one molecule, hence its predictable failure [147].

The nonspecific nature of blood purification therapies makes them particularly interesting from a pathophysiological point of view, since they eliminate mediators only by distinguishing according to their type (endotoxin, patterns, cytokines, or microorganisms) or size [148].

There are several theories that explain the benefits of these therapies. One of the hypotheses is based on the influence of the “peak concentration hypothesis”, in which control occurs by removing cytokines from the circulation. In another (“cytokinetic theory”), it is proposed that the removal of cytokines creates a gradient between blood and tissues [149]. However, these theories, although partly valid, do not fully explain the pathophysiology of blood purification.

We find the view of Honore et al. more interesting; they argued that the reason for this approach is to achieve an “immune homeostasis”, which theoretically reduces the potential damage caused by dysregulation of the host’s response to infection [149].

Other theories, such as the “tip of the iceberg” theory, or the very similar “threshold immune modulation theory”, postulate that the blood levels of mediators are the product of interstitial space and cell compartment saturation, but also of the capacity and kinetics of elimination. Thus, isolated levels of mediators provide an idea of the situation, but perhaps not the overall nature of the process [150].

Given the current limited knowledge of the pathophysiology of the immune response to sepsis, as well as the true capacity of absorption therapies, an attempt can be made to develop a theory that unifies Kumar’s theory and sequential purification therapies [139].

The severity of the infection and the cause of whether or not it causes sepsis is directly related to the bacterial load and its control. If this bacterial load, and therefore the production of endotoxin, is not controlled, the immune system enters a vicious circle. The release of mediators is unacceptable from the point of view of life support, and shock becomes refractory, losing the ability to manage with hemodynamic coherence. In this way, the state of shock becomes irreversible, leading to death, or failing that, to permanent organ failure, and a chronic patient in intensive care with a long-term stay.

Therefore, focus control, resuscitation and phenotype-based life support, optimal antimicrobial therapy, and immunomodulatory therapies such as sequential blood purification are key to the successful treatment of complex patients in septic shock.

### 13.3. Types of Blood Purification Therapies

There are several types of purification therapies, although we focus more on those that we consider specific to adsorption and that can be used in a sequential and phenotypic approach strategy [136].

Convection therapies and their usual filters can remove inflammatory mediators, are limited by pore sizes up to 30 kDa, and can be useful, but the usual pace of recommended renal replacement therapies would be insufficient. Since Ronco’s classic work in which higher doses of convection improved outcomes, all kinds of elevated convection patterns have been used [151].

The rationale is based on the fact that if convection with normal hemofiltration membranes removes cytokines, upon increasing that convection, more are eliminated. However, this hypothesis has not been supported in clinical trials. The most important trial was IVOIRE, which compared two doses of convection (70 mL/kg/h vs. 30 mL/kg/h), with no significant differences [152].

The usual recommended practice is 35 mL/kg/h at the onset of sepsis, although there are groups that continue to postulate the dose of 45–50 mL/kg/h, mainly in refractory shock when the use of adsorption cartridges is not available. The risk of using high doses is the occurrence of dialytrauma associated with the loss of important molecules, such as phosphorus, which delays the withdrawal of mechanical ventilation, and others [153].

There are other modalities of therapy such as using convection cartridges that allow for the elimination of substances with greater molecular weight and that work well by modulating inflammation but eliminate excess nutrients and other valuable molecules, so their use today is anecdotal.

Plasmapheresis and sequential therapies with an adsorption cartridge prior to convection also show partially favorable results, but given their complexity or inexperience in use, they are not frequently used in clinical practice [154].

Specific adsorption therapies use membranes with the ability to retain and/or eliminate molecules directly involved in the pathogenesis of sepsis. These membranes do not necessarily have the possibility of hemofiltration or plasmapheresis, although they can also have it, as in the case of the OXIRIS^®^ membrane. They are, for the most part, membranes aimed at removing endotoxin, molecular patterns, and cytokines, or both. They are examined in detail in the next section.

– Membranes that modulate the immune response to bacteria, mainly eliminating the endotoxin or the bacteria themselves.
– Membranes that modulate the inflammatory response generated by the cells of the immune system, mainly by eliminating molecular patterns of damage and cytokines.
– Membranes with both endotoxin removal possibilities, molecular standards, and cytokines.

### 13.4. Membranes That Modulate the Immune Response to Bacteria (Endotoxin/Bacteria)

The endotoxin is the lipid A of the lipopolysaccharide of the GNB membrane. Its removal could prevent or attenuate the immune system’s response.

The measurement of endotoxin is important to assess the adsorption capacity of different therapies, something that is not entirely clear in the literature. It also makes it possible to identify phenotypes and determine the indication for therapy, and indicate the severity of the infection.

There are several methods for measuring endotoxin, which is very complicated. The most accepted, fastest, and most standardized is the “Endotoxin activity assay”. It is a bioassay based on neutrophil activation by opsonized immune complexes of lipopolysaccharide complement, which allows for the specific detection of the lipopolysaccharide lipid A epitope in a rapid whole-blood assay format. It is a simple and quantitative method that obtains a result within 20 to 30 min [155,156,157].

A direct and linear relationship between elevated endotoxin levels and the severity of sepsis has been demonstrated. Endotoxin levels measured using the endotoxin activity assay (EAA) less than 0.3 are considered normal. An EAA result between 0.3 and 0.6 is a gray area ranging from a normal, tolerable immune response to sepsis. An EAA result above 0.6 indicates what is considered an unacceptable endotoxin load that causes shock and organ failure. An EAA result above 0.9 cannot be measured accurately and is considered a burden that cannot even be eliminated, at least with the strategies employed so far in published studies [144].

The original study that highlighted the value of prognostic measurement of AAS levels was the MEDIC study, which enrolled more than 800 patients admitted to intensive care with suspected sepsis [158].

It is very interesting to review this study. On the day of ICU admission, 57.2% of patients already had AAS levels greater than 0.4, with mortality being 13.2% at levels between 0.4 and 0.6, and 16.8% if it was greater than 0.6. This linear relationship associated with greater severity and mortality with levels of 0.6 in this study is what led to levels of 0.6 being considered high and used as the threshold in the trials that have been carried out and that are being carried out with adsorption therapies. However, there are other studies that have placed this intermediate EAA zone of 0.4–0.6 as also susceptible to treatment, as it can also be associated with septic shock, and with significant severity. The ideal goal of an endotoxin removal treatment would be to lower endotoxin levels, if possible, to less than 0.4 EAA.

The endotoxin levels measured using EAA provide the best direct estimate of endotoxin reduction effectiveness using purification devices. A linear correlation of EAA levels with the amount of polysaccharide, and therefore of bacterial load and severity of infection, has been confirmed, both experimentally and clinically [156].

However, the possibility of endotoxin measurement is not yet available in the vast majority of hospitals. For this reason, “substitutes” have been sought that can help decide the indications for blood purification devices. Infection with GNB is one of them, especially intraabdominal infection, since, although it is polymicrobial, it is essentially caused by GNB. However, MEDIC and other studies described a significant elevation in endotoxin, even when the infection was with Gram-positive bacilli (GPB). This is explained by the bacterial translocation associated with the alteration of intestinal permeability that leads to the patient’s serious situation. Therefore, although isolation of GNB would be a primary indication, any syndrome of high severity can lead to the release of endotoxin at EAA levels greater than 0.4.

It is interesting to know the dynamics of some more readily available markers that can help in the indications and dynamics of adsorption therapies, such as procalcitonin (PCT) and IL6, among others. When inflammation is present, PCT is primarily produced by two mechanisms: (1) Directly induced by lipopolysaccharide (LPS) and other PAMPs. (2) Indirectly by cytokines such as IL-6 and TNF-alpha [159].

Therefore, when a GNB infection occurs, the procalcitonin value is much higher, reaching significantly higher levels than when produced by other microorganisms [160]. In multiple endotoxin adsorption studies, procalcitonin has been assessed as a biomarker of evolution [157].

IL-6 helps in measuring the cytokine response triggered by endotoxin by binding to TLR4. The timing of each biomarker increase is also different. IL-6 initially increases as a response of the innate immune system to injury or infection, and its plasma concentration rises and falls rapidly [116,161].

### 13.5. Adsorption Systems That Remove Endotoxin

#### 13.5.1. Polymyxin Membranes—TORAYMYXIN™

Extracorporeal hemoperfusion with polymyxin cartridges/filters (PMX) such as TORAYMYXIN™ is the most well-known endotoxin removal medium. It is the most widely used in clinical practice to date, especially in Japan, where it has been marketed since 1994. This selective endotoxin removal column contains fibrous adsorbents covalently bound to polymyxin B [162].

Polymyxin B is a polycationic antibiotic that binds to the lipid A portion of the endotoxin and neutralizes its toxicity. Lipid A is the toxic residue of the endotoxin with a conserved structure between GNB species and strains. Therefore, polymyxin B as a ligand is expected to bind to many types of GNB endotoxins. Intravenous use of polymyxin B is contraindicated due to its nephrotoxicity and neurotoxicity; hence, its use as a ligand on the surface of a fibrous material. Extracorporeal hemoperfusion with PMX (PMX-HP) works to adsorb endotoxin from circulating blood.

The dose of endotoxin that it withdraws varies according to the publications. As earlier discussed, perhaps the best means of estimation, the most objective, is the reduction in endotoxin levels, measured in EAA units. In this sense, its effectiveness has been demonstrated in numerous in vitro studies, in animals, and in observational studies and clinical trials [163].

The therapy is usually used with two cartridges, performing two hours of hemoperfusion with each cartridge for two days in a row. The recommended blood flow for polymyxin cartridges is 80 to 120 mL/min. These polymyxin membranes clearly eliminate endotoxin, and as mentioned earlier, there has been an abundance of observational and clinical practice evidence in Japan, but it has not been used in Europe. In 2005, Vincent conducted a small pilot trial in postoperative abdominal sepsis, but using only one cartridge per patient, finding no significant differences, but determining its safety [164].

It was not used in Europe until the publication of a meta-analysis by Dina Cruz, analyzing the published evidence of value, mostly Japanese, which was favorable in terms of improvement in organ failure and survival [165].

This prompted a clinical trial to be conducted in Italy, the EUPHAS trial [166]. In this trial, patients with postoperative abdominal sepsis were recruited within the first 6 h after surgery. They were randomized to receive conventional medical therapy for sepsis vs. hemoperfusion with polymyxin. The most widespread strategy in Japan was used, namely a cartridge two hours a day for two consecutive days, in the first six hours after surgery in patients with postoperative sepsis of abdominal origin. This trial was favorable in terms of mortality to the use of adsorption hemoperfusion with polymyxin cartridges and definitively opened the doors to its use in Europe. This trial was criticized for having few patients (64 patients) and was terminated in the first interim analysis by the ethics committee, as it was very favorable to the intervention arm. Endotoxin was not measured, as they were postoperative abdominal surgery patients with elevated endotoxin levels.

Following this trial, further trials were conducted comparing this therapy with conventional treatment. The first was a multicenter study in France in 2015, ABDOMIX, similar in design to EUPHAS, which included 243 patients with septic shock within the first 12 h after emergency surgery, and failed to demonstrate any benefit with polymyxin cartridge hemoperfusion therapy [167].

Another very important trial in this field was the American EUPHRATES trial, which measured endotoxin and chose an EAA threshold to initiate therapy. Their rationale was to “enrich” the target population by only including patients who definitely had elevated endotoxin levels (EAA ≥ 0.60) so as to increase the chances of success [168].

This study deserves additional explanation: For a patient with sepsis to be randomized, he or she had to have an AAS level greater than 0.6. This threshold was used because the patients with the worst prognosis in MEDIC were those with >0.6 AAS. The polymyxin strategy was the same as in the previous trials and based on the Japanese experience, with two cartridges, with 2 h of hemoperfusion, 2 days in a row, separated by 24 h between cartridges. No differences in mortality were detected between the two groups, but nevertheless, the trial showed that in some patients with septic shock, the burden of endotoxin activity was very high (AAS ≥ 0.9).

This could mean that in selected patients (AAS ≥ 0.90), the burden of endotoxin activity may be too high, and they may be less likely to respond to a standard regimen of two PMX treatments, which would be unable to remove enough endotoxin to improve outcomes.

For this reason, a post hoc analysis was performed to evaluate the impact of the use of this strategy (two polymyxin cartridges) in patients with EAA levels between 0.6 and 0.89 [169]. It was observed that in these patients, treatment with polymyxin significantly decreased mortality. AAS decreased by 12.9%, and mortality was lower in patients with the greatest decrease in endotoxin levels.

Therefore, these results, assessed in the context of personalized medicine, led to the recommendation of the use of the polymyxin cartridge in patients with septic shock with EAA levels between 0.6 and 0.9 [170].

Other meta-analyses after EUPHRATES do not have results favorable for the use of polymyxin with respect to decreased mortality. That is why the Surviving Sepsis guidelines, in the latest update, do not recommend its use (“suggest against using polymyxin B hemoperfusion”), with that argument and that of inequity. However, the latest published meta-analyses recognize the improvement in survival in patients with APACHE < 25, i.e., less severe, i.e., perhaps with lower endotoxin levels [171]. Likewise, the recommendations of many experts are inclined toward the interpretation of the post hoc analysis of EUPHRATES, or, as seen below, for a rethinking of the way of using it, also based on the phenotypes and kinetics of the endotoxin.

#### 13.5.2. ALTECO^®^ LPS Adsorber Membrane

ALTECO^®^ is another hemoperfusion membrane that removes endotoxin from the blood during its passage through the device, thanks to its adsorption technology.

This product contains resin discs (microporous polyethylene) coated with a unique synthetic peptide. This nontoxic peptide is custom-designed to bind to lipopolysaccharides (LPS) with high affinity. It is European, and as such, although it has studies behind it, it does not have as much use as polymyxin. The adsorption capacity of ALTECO^®^ is 7–8 mg LPS, which corresponds to 10 times the amount of endotoxin in a patient’s plasma with an LPS concentration of 1 EU/mL [172].

ALTECO^®^ has been shown to significantly eliminate endotoxin in several studies, such as the one by Ala-Kokko^®^ et al. describing a significant decrease in endotoxin measured using an EAA [173].

Regarding the published clinical use of this membrane, a study that compared its efficacy in septic patients of cardiac surgery, comparing its efficacy with that of polymyxin, using both cartridges for 2 h, two days in a row, stands out. Both ALTECO^®^ and polymyxin decreased procalcitonin levels and endotoxemia, measured with the limulus amebocyte lysate (LAL) test system (ALTECO^®^ from 1.44 U to 0.03 U, and polymyxin from 1.44 U to 0.27 U) [174].

Despite having demonstrated improvement in hemodynamics and organ failure in patients with septic shock in the postoperative period of cardiac surgery, ALTECO^®^ has not been able to demonstrate improved survival. This is probably explained by the low levels of endotoxin in the patients recruited into the trials or by the difficulty in recruiting patients.

Reviewing the most relevant ALTECO^®^ studies in sepsis, the Asset Study stands out, an ambitious European clinical trial that had many problems due to delays in recruitment, which harmed the trial [175]. In addition to the need to have septic shock, and that the intervention should be within the first 12 h, the requirements for patient inclusion in this trial included an SOFA greater than 10, i.e., a significant severity. Like polymyxin, the protocol was two 2 h sessions separated by 24 h. Endotoxin levels were determined using the limulus amebocyte lysate assay method. Endotoxin levels were very low in both groups, as was mortality, which may explain the absence of differences in outcomes between the two groups.

ALTECO^®^ eliminates endotoxins, and therefore can be an alternative used in isolation or in combination with other devices to intervene at this level of sepsis pathophysiology.

#### 13.5.3. Seraph^®^ 100

Seraph^®^ is a promising device that directly removes bacteria from the blood. It has a layer of heparan sulfate, which tricks microorganisms by appearing as the receptors they bind to, and through this binding, it manages to eliminate them. It can absorb many types of bacteria, including those most significant in intensive care infection. There are still not many studies and they have not achieved significant results. The filter removes bacteria, fungi, and viruses, and also appears to remove DAMPs [176].

### 13.6. Membranes That Modulate the Inflammatory Response Generated in the Immune System, Mainly by Eliminating Molecular Patterns of Damage and Cytokines

#### 13.6.1. CYTOSORB^®^

Its total surface area is 45,000 m^2^, and it eliminates molecules of up to 60 kDa (thus not eliminating albumin), which include numerous cytokines, DAMPs, myoglobin, bilirubin, and certain drugs. It has been used in numerous clinical scenarios such as sepsis, COVID-19, and cardiac surgery. In sepsis, it can act on the inflammatory phase by modulating the response, as it has shown great capacity to eliminate cytokines and DAMPs. CYTOSORB^®^ can produce hemodynamic and organ improvement. However, as with all blood clearance therapies, both published trials and meta-analyses have not been able to demonstrate a survival benefit to date, which, together with its high cost, means that its use is not recommended in guidelines [177].

#### 13.6.2. JAFRON Medical

The JAFRON hemoperfusion cartridge, like CYTOSORB^®^, has great capacity to eliminate cytokines. The cartridges contain neutral macroporous resin-adsorbing beads made of a styrene–divinylbenzene copolymer. It eliminates the cytokines most common in sepsis and in the inflammatory states of critically ill patients [178].

### 13.7. Membranes with Endotoxin-Scavenging Capabilities, Molecular Patterns, and Cytokines

#### OXIRIS

The OXIRIS (Baxter) hemofiltration membrane is the only one on the market with the ability to adsorb both cytokines and endotoxin. This, together with the renal replacement function and its antithrombogenic properties, makes it unique in that it brings together four important functions in a single device [179].

**Figure 9:**
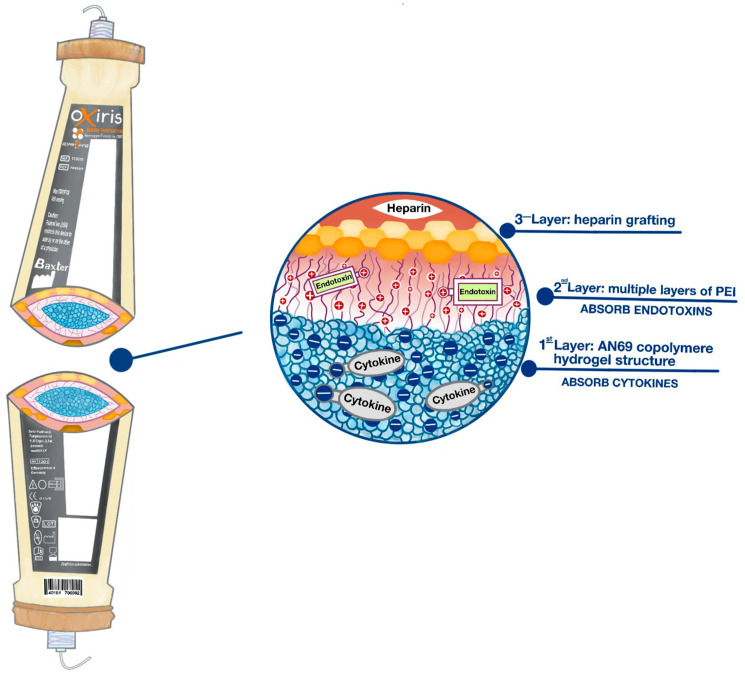
OXIRIS membrane and its cytokine and endotoxin adsorption mechanism.

OXIRIS is an evolution of the AN 69-ST membrane, which in turn was an evolution of AN69. OXIRIS is made up of three different layers [143]:

The central layer is shared by these membranes. It is a hydrogel made up of acrylonitrile and methallyl sulfonate molecules. Thanks to this structure, with a powerful negative charge, the adsorption capacity of cytokines is obtained by binding to their cationic residues. A noteworthy feature of OXIRIS is that, in addition to the pro- and anti-inflammatory cytokines (IL 1, IL 6, IL8 TNFalpha, IL 10, among others), it also could eliminate a molecule such as high-mobility group box 1 protein (HMGB1), which is one of the most important DAMPs in the pathophysiology of sepsis (Figure 9).

OXIRIS shares with AN69-ST a second layer composed of a polyethanolamide (PEI) coating, but in the case of OXIRIS, this layer is much larger. This coating was added to improve biocompatibility and avoid problems arising from the production of bradykinin [180]. This PEI coating allows for not only endotoxin adsorption but also a coating with a third layer of heparin.

Endotoxin adsorption occurs thanks to a significant number of positively charged free amino groups in PEI, which bind to the negatively charged endotoxin. This capacity is much more important, as OXIRIS has significantly more PEI compared to previous membranes. In experimental studies, up to 66% of endotoxins have been shown to be removed within one hour of therapy [181].

Finally, OXIRIS has a third layer of pre-grafted heparin (4500 IU) that improves its antithrombogenic capacity. This is also very important from an adsorption point of view, as the molecules removed by adsorption tend to clog the pores of the filter, decreasing its effectiveness over time.

OXIRIS’s cytokine and endotoxin adsorption capacity has been proven both in vitro and in vivo and evaluated in several clinical studies. Here, we highlight some of them, serving as an example of clarity for their practical use [182].

Malard et al., in a widely cited study, compared the adsorption capacity of cytokines and endotoxin between OXIRIS, CYTOSORB^®^, and TORAYMYXIN™ in vitro. They concluded that OXIRIS had an endotoxin adsorption capacity close to that of TORAYMYXIN™, and cytokine adsorption capacity close to that of CYTOSORB^®^. Endotoxin adsorption in 6 h was 6.9 g with OXIRIS vs. 9.7 g with TORAYMYXIN™ [182]. Considering that it has been reported that the plasma load of endotoxins in the blood of septic patients is in the range of 3 g to 30 g, these results confirm the efficacy of endotoxin clearance with possible clinical outcomes [183].

Until recently, clinical studies were few, but in recent years, the number of quality observational studies and the implementation of clinical trials and clinical use have increased [184].

The first clinical trial was published in 2019 by Broman et al. in patients with GNB sepsis and an endotoxin level greater than 0.03 EU/mL. An improvement was found with OXIRIS in cytokine decrease, SOFA scale, and norepinephrine need. Even with this positive result, the small sample due to recruitment problems (16 patients) means that its conclusions cannot be extrapolated despite the fact that it is a randomized clinical trial [185].

Similar results were also obtained in 2019 by two European observational studies, with a higher number of patients than the Broman trial, also in GNB sepsis. These studies were the beginning of an interest in the investigation of OXIRIS clinical practice results, exemplified by studies looking for mortality results and obtaining mixed results, but with a positive trend [186,187].

In this regard, it is worth highlighting the study by Xie et al. in which they recruited 76 patients with sepsis, mainly by GNB, but also by GPB and fungi, and which demonstrated a decrease in mortality in the OXIRIS group. In the study by Guan et al., with the largest number of patients recruited (176 patients), a significant reduction in short-term mortality (<14 days) was observed [188,189].

OXIRIS has also been used in COVID-19 infection, such as in the study by Gianluca Villa, which aimed to reduce inflammation and organ dysfunction parameters, as well as expected mortality [190]. It has also been used in heart–lung bypass to decrease associated inflammation [191].

Recently, studies on the use of OXIRIS in pediatric sepsis and in sepsis in hematological patients have been published [192,193].

Numerous studies are underway, including several clinical trials with different objectives, the results of which will be available in the next two years.

In studies of the efficacy of new therapies in intensive care, it is difficult to obtain decisive results in mortality, but it is important to demonstrate results with a large group of studies. The available studies on OXIRIS had about 100 patients per study and showed that it has the ability to decrease the levels of cytokines, endotoxin, and related biomarkers such as procalcitonin, in addition to improving hemodynamic parameters and reducing the need for vasopressors [194].

An initiative of great interest is the Global ARRT International Registry (NCT03807414, official website: www.arrt.eu, accessed on 9 September 2022), which is an international registry of the clinical use of OXIRIS, in which the use of other membranes and other adsorption devices is also recorded. Its results, being worldwide, facilitate the possibility of making comparisons and indicate not only its efficacy but also its frequency of use and what type of patients it is used on.

The indications and timing of the use of OXIRIS are not yet clearly defined, although two expert recommendations have been published, one European and another from the Asia-Pacific [195,196].

In principle, it seems clear that the early use of OXIRIS could be indicated if the patient with sepsis is associated with renal failure with high amine requirements, hemodynamic instability, or incoherence with poor perfusion due to impaired microcirculation.

The problem may lie in the latest published evidence that very early timing of renal replacement does not seem to improve outcomes in critically ill patients. The dose of renal replacement therapy with OXIRIS appears to be 30–35 mL/kg, and there is an important consensus [195]. There is less consensus on its use alone without the need for renal replacement [196].

The recommended time for its replacement is 12 h in the situation of septic shock, according to the consensus, although it depends on the state of the membrane and the adsorption performed, since its active duration can be up to 72 h. It is difficult to evaluate in each situation, since, if the adsorption is massive, it could jeopardize its effectiveness if the circulating levels of cytokines or endotoxin are very high, with a risk of thrombosis of the filter due to clogging of the pores.

In theory, OXIRIS requires less anticoagulation due to its heparin coating, although the recommendation is to use anticoagulation, which can be at lower doses. Case series have been published with good results of the association of OXIRIS with citrate as an anticoagulant [197].

The effects of dialytrauma secondary to the use of renal replacement therapy and blood clearance on nutrients, phosphorus, and antibiotic therapy should be considered. Care should be taken to ensure that the beneficial effects of cytokine and endotoxin adsorption are not impaired by poor management therapy; homeostasis should always be sought [198].

OXIRIS’ price, and endotoxin and cytokine adsorption capacity, as well as its hemofiltration capacity for both convection and dialysis, make the OXIRIS membrane unique, being the only hemoperfusion cartridge capable of intervening in the three phases described at the beginning of this Section. It can be used alone throughout all three phases, or in combination with other membranes [139].

A systematic review and meta-analysis using the GRADE methodology on the efficacy of OXIRIS on 28-day mortality in sepsis was recently published. This meta-analysis (14 studies/695 patients) shows a significant reduction in mortality (OR) 0.53 and ICU days of patients with sepsis using OXIRIS compared to other filters. In the OXIRIS group, there was improvement in SOFA, decreased doses of norepinephrine, and decreased IL-6 and lactic acid levels. Although the observational studies are good, of medium or high quality, the evidence is of low quality due to the absence of good-quality clinical trials [199].

Our clinical practice and recommendations for ideal OXIRIS use are very similar to the recommendations of the Asia-Pacific group [195]. We recommend its use in shock refractory to tissue perfusion-guided resuscitation [14].

We use it in patients for whom the perfusion has not recovered, either measured by lactate or capillary filling time, and who also have requirements of amines at high doses (noradrenaline at doses >0.5 mcg/kg/min) or with the need to administer two vasopressors. Usually, these patients will already have a degree of multiorgan dysfunction in which acute renal failure is usually present. If the patient meets these criteria, even if there is no clear oliguria and even if KDIGO AKI is equal to one, we start OXIRIS. We can associate OXIRIS with another endotoxin clearance device such as ALTECO^®^ or TORAYMYXIN™ if we are in the first 12 h after diagnosis and hemodynamic shock is more important or organ dysfunction involves a SOFA greater than eight. OXIRIS is replaced every 6–8 h to avoid a decrease in efficacy due to the massive adsorption of cytokines and endotoxins. When hemodynamic parameters improve, and the need for vasopressors, levels of biomarkers such as procalcitonin and NT-proBNP decrease, usually in the first 24–36 h, we move to an OXIRIS cartridge change every 12 h, for the next 48 h (2–4 days), until entering a stabilization phase. The same filter can then continue to be used to improve congestion in the evacuation phase of the ROSE or for organ support of renal failure if it persists [35].

It would be ideal to be able to integrate the monitoring of IL-6 levels for cytokines, as well as endotoxin levels, to assess the indication and the evolution of its efficacy. For example, in the case of endotoxin with EAA levels less than 0.3, it would not be of interest, and with EAA levels above 0.9, another endotoxin adsorption device would surely have to be added [200].

### 13.8. Sequential or Synergistic Therapies with OXIRIS Adsorption Therapies

The pathophysiology of sepsis has several different moments at which adsorption therapies can be enacted. Current treatment of sepsis is based on focus control, when possible; antibiotic treatment; and hemodynamic resuscitation. Even if all these measures are carried out early, if the bacterial load threshold is exceeded, and sepsis is triggered, apart from life support, only adsorption therapies appear as a reasonable alternative for rescue.

It can be operated at five levels, in chronological order and sometimes synergistically and simultaneously, with the same machine and different devices, with less invasiveness [139]:

1. Removing pathogens directly from the circulation, as with Seraph^®^.
2. Eliminating PAMPs (the most emblematic endotoxins), as with TORAYMYXIN™, ALTECO^®^, and OXIRIS.
3. Eliminating DAMPs (the most representative being HMGB1), as with OXIRIS, CYTOSORB^®^, and SERAPH, among others.
4. Eliminating cytokines (interleukins and TNF-alpha, among many others) and immunomodulating the secondary response to microorganisms and PAMPs and DAMPs, as with OXIRIS, CYTOSORB^®^, and JAFRON.
5. Extracorporeal organ support, mainly renal, but increasingly respiratory and cardiological.

**Figure 10:**
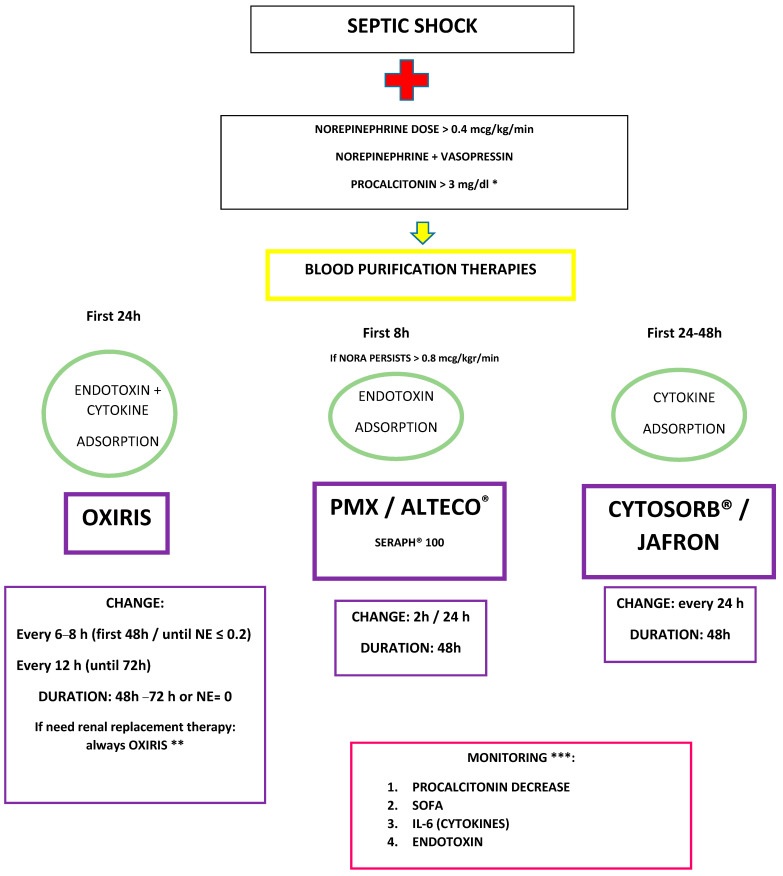
The different techniques of blood purification are a clinical choice in the patient with septic shock that depends on context, clinical situation, and expertise. This figure is intended to be a cognitive aid rather than an algorithm. NE: norepinephrine; PMX: polymyxin. * Procalcitonin is suggested as an endotoxin surrogate for evolution. ** OXIRIS is the only membrane with adsorption and renal clearance capacity at the same time, so our recommendation is always to use it in this situation, regardless of the decision or not to associate more devices. *** IL-6 and Endotoxin only if you can do it.

Figure 10 shows a proposed decision-making algorithm for patients with septic shock based on clinical situations.

The recommended monitoring depends on the capacity of each center and the context, which reflects the ideal.

**Figure 11:**
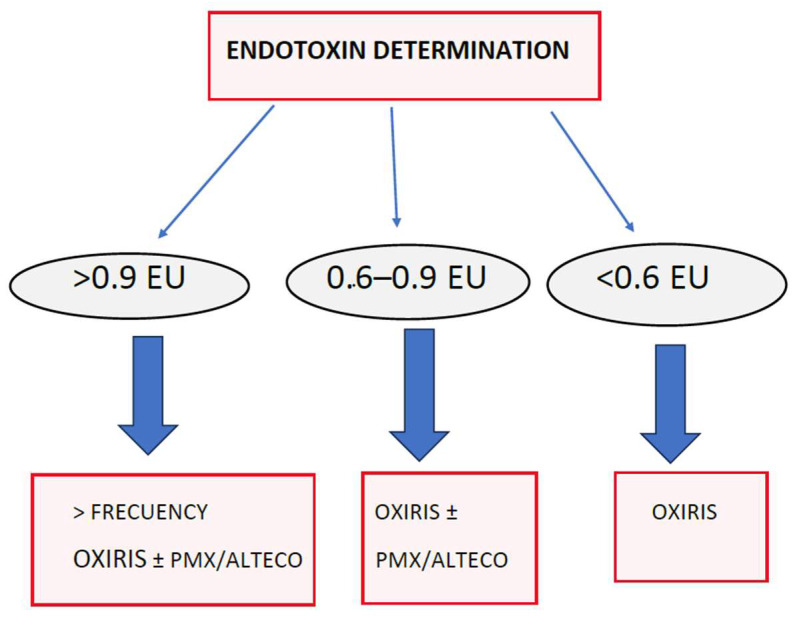
Determination of endotoxin levels is available but is not yet frequently used, although it is highly recommended. Endotoxin levels can help in deciding not only the therapy or devices but also their association and frequency.

There is a need for a marker that will help indicate these therapies at the right time, which will allow for choosing the type of adsorption therapy, as well as its sequence or its use in a synergistic way. We have evidence that patients with EAA levels between 0.6 and 0.9 have better outcomes using an endotoxin removal device such as TORAYMYXIN™ two hours a day for two consecutive days. Also, when endotoxin levels are less than 0.3, the results are not better, and satisfactory results are not achieved, so why is this monitoring not used more? Do we think it is a big deal? (Figure 11).

It is not known whether using the devices more frequently with EAA levels above 0.9, as Ferretti has already published, could improve the results. There is no one-size-fits-all solution. Ferretti et al. proposed a strategy for the use of the PMX cartridge in which daily treatments with PMX cartridges are administered for 2 h until near-normal EAA levels are achieved (<0.40 EAA). This strategy was tested in a study of 17 postsurgical patients with septic shock. Patients with AAS > 0.6 were treated to an AAS level < 0.40. A group of eight patients with AAS > 0.85 required three hemoperfusion sessions, while two patients with a hemoperfusion of 0.99 required four hemoperfusion sessions. All patients survived more than 28 days [201].

In this regard, Iwagami et al. used a paired propensity score statistical technique to evaluate the use of PMX patients with septic shock who were receiving continuous renal replacement therapy. Mortality was significantly lower for the PMX group. Patients who received two sessions of PMX had lower mortality than those who received only one session, indicating a possible “dose–response” [202].

It is also not known whether using two different types of devices can complement each other, as we have suggested with the use of combinations with OXIRIS.

For example, two different endotoxin scavenging devices could be used in patients with high endotoxin levels, hemodynamic instability, and poor peripheral perfusion, always assessing cost-effectiveness. Likewise, it may occur that patients with low endotoxin levels but high markers of inflammation benefit only from a clearance with OXIRIS that combines both, or from a clearance only of cytokines. It is about applying a personalized rational essential therapy [139].

It is a therapy with a clear pathophysiological basis, with increasing evidence of improvement in important outcomes, although not yet clearly in mortality, and the technology is improving rapidly. We believe that there is still much to investigate and discover in this area in the coming years [203,204].

## 14. Conclusions

Patients with septic shock pose a challenge for us because of their high mortality. We have witnessed many changes and debates in the diagnosis and treatment of our patients over the past 25 years [205]. This review aimed to review some cutting-edge treatment possibilities and provide an overview of the approach to the most at-risk septic patients. In the following points, we summarize the present and near-future sepsis challenges that generate debate and that we have dealt with in this narrative review.

1. The challenges related to sepsis have to do with the balance between guidelines and evidence, with a personalized approach and in certain contexts. The high mortality rate of septic shock obligates us to explore cutting-edge therapies.
2. Sepsis is a time-dependent pathology, and it is necessary to implement sepsis care programs to improve outcomes.
3. Hemodynamics is directed toward personalized medicine and the recognition of different phenotypes to discover the timing of the initiation of vasopressor treatment and the appropriate dose of fluid therapy. The evaluation of perfusion, for example, with the capillary refill time, is key in clinical decision making.
4. At present, the focus is not only on the initial timing but also on the so-called “deresuscitation”, with the early weaning of support therapies and targeted elimination of fluids. The novel concept of fluid tolerance is the new framework.
5. Cardiac POCUS is a useful tool for the diagnosis and characterization of shock, the detection of myocardial dysfunction associated with sepsis, and for guiding fluid therapy in patients with sepsis.
6. Blood purification therapies, mainly those aimed at eliminating endotoxins and cytokines, are becoming attractive in the early management of patients in septic shock. Sequential therapy of endotoxin and then cytokine removal with different devices; in parallel, eliminating both at the same time; or with the same purification system that eliminates both without the need for two devices, are promising strategies in certain phenotypes and with appropriate timing.

## Figures and Tables

**Table 1:** Echocardiographic phenotypes of septic shock. Obtained from Geri et al., 2019 [129]. ∆SVC: superior vena cava collapsibility index; AUC: area under the curve; CO: cardiac output; DAP: diastolic arterial pressure; E/e’: ratio between early mitral inflow velocity and mitral annular early diastolic velocity; HR: heart rate; ICU: intensive care unit; LVEF: left ventricular ejection fraction; LVFAC: left ventricular fractional area of change; NPV: negative predictive value; PaO_2_/FiO_2_: arterial pressure of oxygen-to-fraction of inspired oxygen ratio; PPV: positive predictive value; RV/LV EDA: right ventricular-to-left ventricular end diastolic areas; SAP: systolic arterial pressure; ScVO_2_: central venous oxygen saturation TEE: transesophageal echocardiography; VTI: velocity time integral.

Phenotype (% of Subjects in the Sample)	Phenotype Features	Ultrasound and Hemodynamic Findings	Diagnostic Performance	Mortality	Remarks
**Cluster 1** **Well-resuscitated (16.9%)**	Normal systolic function of both ventricles Nonfluid responsiveness	Normal TEE		7 days: 9.8% ICU: 21.3%	
**Cluster 2** **LV Systolic dysfunction (17.7%)**	Decreased LVEF and LVFAC Low CO High lactate and high doses of noradrenaline Nonfluid responsiveness	LVEF < 40% Aortic VTI < 14 cm LVFAC < 33%	Sensitivity: 54.7%% Specificity: 97.6% PPV: 83.3%% NPV: 90.9%% AUC: 0.959	7 days: 32.8% ICU: 50%	14% of patients had LVEF > 45% ScVO_2_ normal E/e’ normal
**Cluster 3** **Hyperkinetic state (23.3%)**	Elevated LVEF High CO Nonfluid responsiveness	Aortic VTI > 20 cm HR: < 106 LPM LVFAC > 56%	Sensitivity: 17.9%% Specificity: 98.2%% VPP: 75%% NPV: 79.7%% AUC: 0.885	7 days: 8.3% ICU: 23.8%	
**Cluster 4** **RV failure (22.5%)**	High RV/LV EDA LVEF normal or supranormal Nonfluid responsiveness	RV/LV EDA > 0.8 SAP < 100 mmHg DAP < 51 mmHg	Sensitivity: 29.6%% Specificity: 98.9%% PPV: 88.9%% NPV: 82.9%% AUC: 0.951	7 days: 27.2% ICU: 42%	Increased number of patients with PaO_2_/FiO_2_ < 200 mmHg
**Cluster 5** **Still hypovolemic (19.4%)**	Low CO Elevated LVEF Fluid responsiveness	Aortic VTI < 16 cm E wave < 67 cm/s ∆SVC > 39%	Sensitivity: 25.7%% Specificity: 99.3%% VPP: 90%% NPV: 84.7%% AUC: 0.955	7 days: 23.2% ICU: 38.6%	These patients had received more volume in the initial stages of resuscitation compared to the other phenotypes

## Abbreviations

Acute kidney injury (AKI), Acute respiratory distress syndrome (ARDS), American Society of Echocardiography (ASE), Area under the curve (AUC), “Code Sepsis” (CS), Cardiac output (CO), Capillary refill time (CRT), Central venous oxygen saturation (SvcO_2_), Central venous pressure (CVP), Danger-associated molecular patterns (DAMPs), Diastolic arterial pressure (DAP), Endotoxin activity assay (EAA), European Society of Intensive Care Medicine (ESICM), Left ventricular ejection fraction (LVEF), Gram-negative bacteria (GNB), Gram-positive bacilli (GPB), Heart rate (HR), Inferior vena cava (IVC), Intensive care unit (ICU), Left ventricle (LV), Left ventricular outflow tract (LVOT), Mean arterial pressure (MAP), Mitral annular plane systolic excursion (MAPSE), National early warning score (NEWS), N-terminal pro B-type natriuretic peptide (NT-proBNP), Norepinephrine (NE), O_2_ delivery (DO_2_), O_2_ consumption (VO_2_), Pathogen-associated molecular patterns (PAMPs), Point-of-care ultrasound (POCUS), Princess Sepsis Code (PSC), Pulse Pressure Variation (PPV), Randomized controlled trail (RCT), Reactive oxygen species (ROS), Right ventricle (RV), Septic cardiomyopathy (SCM), Systolic arterial pressure (SAP), Stroke volume variation (SVV), Superior vena cava (SVC), Surviving Sepsis Campaign (SSC), Tumor necrosis factor (TNF), Transthoracic echocardiography (TTE), Transesophageal echocardiography (TEE), Velocity time integral (VTI), Vasopressin (AVP), Pulmonary artery catheter (PAC), Diastolic shock index (DSI), High-mobility group box 1 protein (HMGB1), Interleukin-1 (IL-1), Polyethanolamide (PEI), quick-SOFA (qSOFA), Tissue red blood cell perfusion (tRBCp), Venoarterial carbon dioxide gradient (DpCO_2_), Venous excess Doppler ultrasound (VExUS).
